# Stigmasterol from Pluchea indica Leaves Reduces Spermatogenesis and Alters Sperm Morphology in Male Rat: In Vivo and In Silico Study

**DOI:** 10.12688/f1000research.180671.1

**Published:** 2026-05-14

**Authors:** Poncojari Wahyono, Kiky Martha Ariesaka, Eko Susetyarini, H. Husamah

**Affiliations:** 1 Department of Biology Education, Universitas Muhammadiyah Malang, Malang, East Java, Indonesia; 2 Department of Medicine, State University of Malang, Malang, East Java, Indonesia

**Keywords:** Sperm Morphology, Pluchea indica, Rattus norvegicus, Spermatogenesis, Stigmasterol, In Silico Network Pharmacology

## Abstract

**Background:**

Limited male contraception choices have prompted the hunt for natural antifertility drugs. This experimental study investigates the antifertility effects of stigmasterol extracted from *Pluchea indica* leaves in male rats ( *Rattus norvegicus*).

**Methods:**

This was a randomized experimental study involving twenty-four male rats, which were randomly allocated into four groups (n = 6/group): one control group and three treatment groups receiving stigmasterol at 0.25, 0.5, and 0.75 mg/kg body weight orally for 45 days. Histological examination of testicular tissue and evaluation of sperm morphology were performed to assess spermatogenic activity. To elucidate possible molecular mechanisms, an in silico network pharmacology analysis was conducted using SEA, CTD, DAVID, STRING, and Cytoscape platforms.

**Results:**

A significant reduction in spermatogenic cell counts and increased sperm morphological abnormalities were observed, particularly at the highest dose (0.75 mg/kg BW) (p = 0.043). Network pharmacology analysis identified 76 predicted protein targets related to spermatogenesis, hormonal regulation, and inflammation, including EGFR, IL6, TNF, ESR1, and AKT1.

**Conclusions:**

This experimental study demonstrates that stigmasterol from *P. indica* leaves, particularly at 0.75 mg/kg BW, exerts antifertility effects by reducing spermatogenic cells and inducing sperm abnormalities in male rats. In silico findings suggest that stigmasterol may influence key molecular pathways involving hormonal balance, cell proliferation, and inflammation, supporting its potential role as a natural male contraceptive agent.

## Introduction

The main problem faced in Indonesia in the field of population is the still high rate of population growth. ^1^ According to data from Statistics Indonesia, the population density in Indonesia in 2022 reached 275,773,800 and in 2023 increased to 278,696,200 people. ^2^ Government efforts to control the rising population are carried out through the Family Planning Program regulated by Law No. 52 Article 1 of 2009. The Family Planning Program offers several contraceptive methods. ^3, 4^ Field conditions show that contraception is more directed at women because options for men are still very limited. Therefore, further research is needed to discover safe contraceptive methods for men. One of the approaches being explored is the use of traditional medicinal plants.

Traditional medicines intended for public use must comply with requirements established in distribution permits and must not pose health risks to users. ^5– 11^ Three main aspects need to be considered for any medicinal product to be distributed: safety, quality, and efficacy. ^12– 14^ The Indonesian Food and Drug Authority Regulation Number 10 of 2022 states that to ensure the safety of any medicinal substance, including herbal medicines intended for humans, preclinical in vivo testing must be conducted, including toxicity and efficacy assessments. ^15, 16^

*Pluchea indica* leaves contain many active compounds, such as alkaloids, flavonoids, tannins, essential oils, sodium, potassium, aluminum, calcium, magnesium, phosphorus, saponins, and steroids. Flavonoids, alkaloids, and tannins have been reported to affect spermatogenesis, testosterone levels, and the number of offspring in female white rats. ^17, 18^ Among the compounds with potential antifertility activity is stigmasterol. ^19^ Stigmasterol belongs to the phytosterol group, which is a derivative of steroid compounds. ^20– 24^

The working principle of stigmasterol is cytotoxic and involves inhibition of hormonal pathways, which can result in decreased testosterone and impaired spermatogenesis. Similar mechanisms of hormonal modulation have been described in other contexts, such as the antiproliferative effects mediated by GnRH receptor signaling pathways. ^25– 28^ Parameters that can be used to assess the quality of the reproductive system include the weight of reproductive organs, sperm abnormalities, sperm count, morphology, and motility. Sperm morphology plays an important role in determining function, especially in the ability to penetrate cervical mucus and interact with the zona pellucida. ^29– 31^ Therefore, sperm shape has an essential role. Previous researches also reported that the assessment of sperm quality includes abnormalities, viability, concentration, and the count of motile spermatozoa. ^24, 32^ Previous studies on male antifertility agents generally focused on these parameters, ^32– 35^ while this study focuses specifically on spermatogenic cell counts and sperm morphology, which remain less explored.

In addition to the in vivo experiments, this research also incorporated in silico network pharmacology analysis to comprehensively predict the molecular targets and biological pathways through which stigmasterol may exert its antifertility effects. This integrative approach is expected to provide mechanistic insights supporting the experimental findings.

## Methods

### Study design

This experimental study was conducted using a completely randomized design (CRD). The treatment period lasted for 45 days. Twenty-four adult male *Rattus norvegicus* aged 5–6 weeks with an average body weight of 200 g were randomly assigned into four groups: one control group receiving distilled water/aquades as the vehicle and three treatment groups receiving stigmasterol isolated from *P. indica* leaves at doses of 0.25 mg/kg BW (P1), 0.5 mg/kg BW (P2), and 0.75 mg/kg BW (P3). Each group consisted of six rats. The study was designed to evaluate the in vivo antifertility effects of stigmasterol from *P. indica* leaves on spermatogenic cells and sperm morphology, supported by network pharmacology analysis.

Each rat was considered an experimental unit. The sample size consisted of six rats per group and 24 rats in total. No formal a priori sample size calculation was conducted; the sample size was determined based on the completely randomized experimental design, previous related preclinical studies, feasibility, and the reduction principle in animal research. Inclusion criteria were healthy and active adult male rats aged 5–6 weeks with an average body weight of approximately 200 g. No animals were excluded from the final analysis. After acclimatisation, rats were randomly allocated into the control and treatment groups. The specific random sequence generation method was not recorded.

### Plant material, extraction, and isolation of stigmasterol

The *P. indica* leaf simplicia used as the source material for stigmasterol isolation was obtained from the Herbal Laboratory of Materia Medika (Lahor Street No.87, Pesanggrahan, Batu City, East Java, Indonesia). Extraction and isolation were performed at the Mark Herb Laboratory, Bandung Institute of Technology, Indonesia (ITB Innovation Park Bandung Technopolis, Bulevar Utama Street No. 3, Cisaranten Kidul, Gedebage, Bandung, West Java, Indonesia). Simple maceration was conducted, followed by solvent evaporation using a rotary evaporator to obtain a concentrated extract. The extract was separated via liquid chromatography, and fractions were purified with column chromatography. Thin-layer chromatography analysis confirmed the presence of stigmasterol. Recrystallization with methanol yielded pure stigmasterol in crystalline powder form.

The isolated stigmasterol was used as the test compound and prepared according to the assigned dose for each treatment group. The compound was diluted in distilled water/aquades immediately before oral administration. The plant material was authenticated by Prof. Dr. Elly Purwanti (a professor of botany at Universitas Muhammadiyah Malang, Indonesia). A voucher specimen with the number 303/LAB-BIO/UMM/2025 was deposited at Botany Section of Biology Laboratory, Universitas Muhammadiyah Malang (Raya Tlogomas Street No. 246, Malang, East Java, Indonesia).

### Reagents and materials

All reagents and materials used in the study were documented, including their purpose, amount or concentration, supplier/manufacturer, and catalogue or internal laboratory reference number. The main test compound was stigmasterol isolated from *P. indica* leaves, while distilled water/aquades was used as the vehicle and control treatment. Isoflurane was used as the anesthetic agent, with oxygen as the carrier gas. Histological processing used 10% neutral buffered formalin, graded ethanol, xylene, paraffin wax, hematoxylin, and eosin. Details of the reagents and materials are provided in Table 1.

**Table 1.** Reagents and materials used in the study.

Reagent/material	Purpose	Amount/concentration used	Supplier/manufacturer	Catalogue/internal laboratory reference number
Stigmasterol isolate from *Pluchea indica* leaves	Test compound	0.25, 0.5, and 0.75 mg/kg BW; prepared according to rat body weight	Isolated at Mark Herb Laboratory, Institut Teknologi Bandung, Indonesia	Not applicable; laboratory-isolated compound
*Pluchea indica* leaf simplicia	Source material for stigmasterol isolation	As required for extraction/isolation	Herbal Laboratory of Materia Medika, Batu City, East Java, Indonesia	Not applicable
Distilled water/aquades	Vehicle and control treatment	As required for oral gavage	CV. Krida Tama Persada	88/LB/FK-UMM
Isoflurane	Anesthetic agent	4–5% for induction; 2–3% for maintenance in oxygen	Dexa Medica	201/LB/FK-UMM
Oxygen	Carrier gas for isoflurane anesthesia	As required	Prima Guna Gas	88/LB/FK-UMM
10% neutral buffered formalin	Tissue fixation	10%	Delta Laboratorium	78/LB/FK-UMM
Ethanol	Tissue dehydration	70%, 80%, 90%, 96%, and absolute ethanol	Supelco	112/LB/FK-UMM
Xylene	Tissue clearing	0.03–0.05 mL, 100%	Srikandi Chemical Supplier	152/LB/FK-UMM
Paraffin wax	Tissue embedding	As required	Delta Laboratorium	79/LB/FK-UMM
Hematoxylin	Nuclear staining	1% for 3 minutes	PT Risky Putra Kasih	301/LB/FK-UMM
Eosin	Cytoplasmic staining	1% for 3 minutes	PT Risky Putra Kasih	302/LB/FK-UMM
Oral gavage needle/sonde	Oral administration	As required	Delta Laboratorium	54/LB/FK-UMM

### Preparation and maintenance of experimental animals

Animals were acclimatized for 7 days in plastic cages (45 cm × 30 cm × 15 cm), with each cage housing three rats. The ambient temperature was maintained at 20–25 °C. Rats received a daily diet equivalent to 10% of body weight and had free access to distilled water. Bedding material (rice husks) was replaced twice weekly.

Animals were monitored daily for general health, behaviour, food and water intake, and signs of pain or distress, including reduced mobility, abnormal posture, severe lethargy, laboured breathing, marked body weight loss, or persistent refusal to eat or drink. Humane endpoints were established to allow early euthanasia if severe distress or deteriorating health occurred. No unexpected adverse events were observed during the study.

All animal procedures were conducted under controlled laboratory conditions and were designed to minimize pain, distress, and unnecessary suffering. The study protocol was approved by Ethics Committee of the Faculty of Medicine, Universitas Muhammadiyah Malang, Indonesia, under approval number No. E54/255/KEPUMM/VIII/2023 on 31/08/2023. The experimental study commenced on 01/09/2023, after the approval had been granted.

This study did not involve privately owned animals. All animals used in this study were laboratory rats obtained for experimental purposes under the approved institutional animal ethics protocol; therefore, owner informed consent was not applicable.

### Treatment administration

Body weights were measured before treatment initiation and prior to dissection using an analytical balance. The assigned doses of stigmasterol were administered orally using an oral gavage. The control group received distilled water/aquades as the vehicle, while the treatment groups received stigmasterol at the assigned doses. Each dose was prepared according to the body weight of each rat immediately before administration to ensure dosing accuracy.

The assigned dose was adjusted according to the most recent body weight of each rat. Stigmasterol was diluted in distilled water/aquades immediately before administration. The preparation was administered once daily by oral gavage for 45 days. The control group received distilled water/aquades using the same route and schedule.

### Anesthesia, Euthanasia, and sample collection

After the treatment period, all rats were humanely handled to minimize stress before tissue collection. The animals were anesthetized using isoflurane inhalation. Anesthesia was induced with 4–5% isoflurane in oxygen in an induction chamber and maintained with 2–3% isoflurane in oxygen until a deep anesthetic plane was achieved. The depth of anesthesia was confirmed by the absence of pedal withdrawal and corneal reflexes.

Under deep anesthesia, the animals were euthanized by cervical dislocation as a secondary physical euthanasia method performed by trained personnel. Death was confirmed by the absence of heartbeat, respiration, and reflex responses before dissection. The euthanasia procedure was conducted in accordance with the approved institutional animal ethics protocol and the principles of the American Veterinary Medical Association Guidelines for the Euthanasia of Animals. ^36^

After death was confirmed, organs including the liver, kidneys, testes) and epididymal spermatozoa were collected. Testes were cleaned and fixed in 10% neutral buffered formalin, processed for paraffin embedding, and stained with hematoxylin-eosin for histological observation 16. Sperm samples were diluted with 0.5 mL TRIS buffer and further diluted 200-fold using 0.9% NaCl solution 10. The suspension was homogenized and prepared on glass slides for evaluation.

### Observation of spermatogenic cells and sperm morphology

Sperm morphology was assessed by mixing the sperm suspension with 1% eosin, covering with a coverslip, and examining under a light microscope at 400× magnification. Morphology was categorized as normal when >50% of spermatozoa were normal 17. Spermatogenic cell counts (spermatogonia, spermatocytes, spermatids) were determined by observing five fields of view under a light microscope at 400× magnification. All observations were documented photographically.

Microscopic observations were conducted using coded slides where possible to reduce observer bias. Uncropped and unedited parent microscopic images were retained as underlying data. Representative cropped images used in the manuscript were prepared only for presentation purposes, while the original parent images were deposited as underlying data in the repository.

### Network pharmacology analysis

The SMILES structure of stigmasterol (PubChem ID: 5280794) was retrieved from PubChem. Two databases were used to predict target proteins: SEA Target ( https://sea.bkslab.org/) with a p-value cutoff <0.05 and Tanimoto coefficient ≥ 0.4, and the Comparative Toxicogenomics Database (CTD) ( https://ctdbase.org/). Target annotations were performed using the DAVID web server ( https://david.ncifcrf.gov/) for Gene Ontology Biological Process enrichment (p < 0.05). Proteins associated with spermatogenesis were retrieved from GeneCards ( https://www.genecards.org/). Protein–protein interaction networks were analyzed using STRING v12.0 ( https://string-db.org/) with Homo sapiens as the organism and a confidence score ≥ 0.7. The TSV output was imported into Cytoscape v3.10 for network topology analysis, including degree and closeness centrality calculations. All databases were accessed on [insert access date]. Input files, predicted target lists, enrichment outputs, STRING interaction files, and Cytoscape network files were retained and deposited as underlying or extended data.

### Data analysis

Data were processed using IBM SPSS Statistics software. Normality and homogeneity tests were performed prior to statistical analysis. One-way ANOVA was used to determine significant differences. When significant, treatments were compared using the Least Significant Difference (LSD) test. The level of significance was set at p ≤ 0.05.

The exact number of animals included in each analysis was six per group. No animals or data points were excluded from the final analysis. Individual animal-level data, values underlying means and standard deviations, values used to generate figures, and statistical output files were prepared for deposition in an open repository.

## Results

### Spermatogenic cell counts

The administration of stigmasterol from *P. indica* leaves affected spermatogenic cell numbers in male rats ( *Rattus norvegicus*). Table 1 shows the mean counts of spermatogonia, spermatocytes, and spermatids across treatment groups. The control group exhibited higher average counts of spermatogenic cells compared to all treatment groups. In particular, the highest stigmasterol dose (0.75 mg/kg BW) resulted in the lowest counts of spermatogonia (24.4), spermatocytes (23.6), and spermatids (21.3).

**Figure 1.**
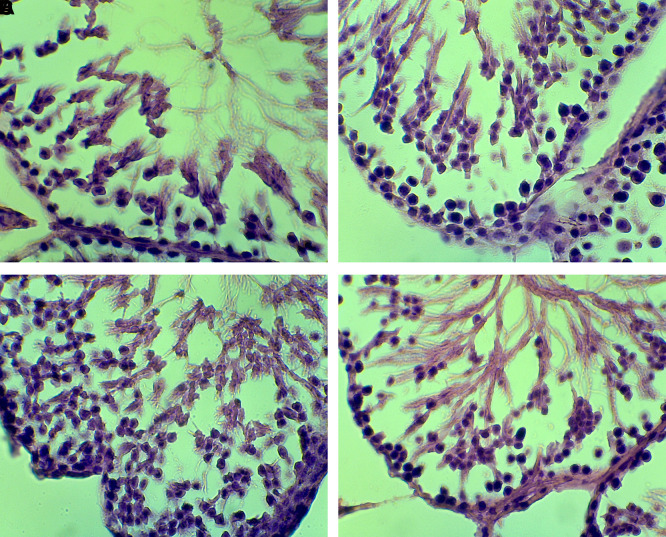
(A) Histology of rat testes stained with hematoxylin-eosin (400× magnification); K: control. (B) Histology of rat testes stained with hematoxylin-eosin (400× magnification); P1: treatment 1. (C) Histology of rat testes stained with hematoxylin-eosin (400× magnification); P2: treatment 2. (D) Histology of rat testes stained with hematoxylin-eosin (400× magnification); P3: treatment 3.

Statistical analysis using One-Way ANOVA revealed a significant difference among groups (p = 0.043). The LSD test indicated a dose-dependent decline in spermatogenic cell counts, with the P3 group showing a significantly lower mean count (23.44 ± 2.26) compared to the control (29.93 ± 3.37) ( Table 1). Representative histological images are presented in Figure 1 **(A-D)**.

### Sperm morphologic

**Figure 2.**
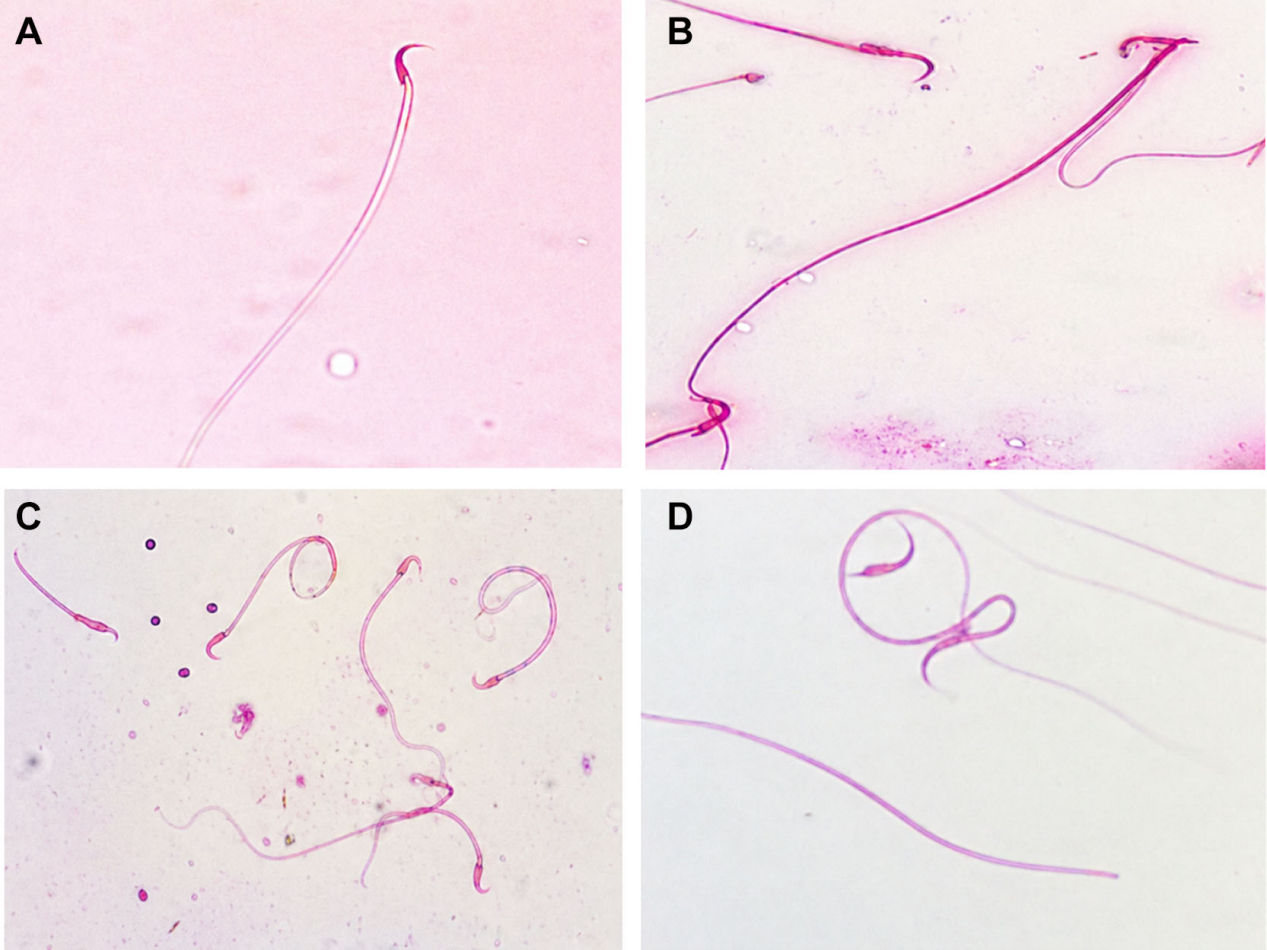
(A) Rat spermatozoa: K = Control group. (B) Rat spermatozoa: P1 = Treatment 1. (C) Rat spermatozoa: P2 = Treatment 2. (D) Rat spermatozoa: P3 = Treatment 3.

Observations presented in Figure 2 **(A-D)** show that white rats possess spermatozoa with normal morphology, consisting of a head, neck, and tail. However, in abnormal spermatozoa, these parts were incomplete and exhibited various structural defects. In the control group, spermatozoa appeared normal, with intact components including the head, neck, and tail. In contrast, the treatment groups showed multiple abnormalities, such as heads lacking an acrosome, headless tails, detached heads, wavy necks, coiled tails, tails forming an angle, and tails without a head.

The highest mean percentage of sperm abnormalities was observed in the P3 group (0.75 mg/kg BW) at 32%. In contrast, the control group showed the lowest mean abnormality percentage, with only 12%. Regarding the percentage of normal spermatozoa, the highest mean was found in the control group. Normality and homogeneity tests, followed by One-Way ANOVA and Least Significant Difference (LSD) tests, were performed on the data. The One-Way ANOVA test indicated a significant difference among treatment groups (p = 0.00).

As shown in Table 2, significant differences were found among treatments in both normal and abnormal spermatozoa counts. In the normal sperm group, P3 (mean 68%) showed a significantly lower percentage compared to the other groups. In the abnormal sperm group, P3 (mean 32%) had a significantly higher percentage than the control, P1, and P2, which had mean values of 12%, 13%, and 14.83%, respectively.

**Table 2.** Effects of Stigmasterol from *Pluchea indica* Leaves on Spermatogenic Cells, Sperm Morphology, and Sperm Dimensions in Male Rats.

Parameter	Group (Dose)	Mean ± SD (Spermatogenic Cell Counts)	Mean Percentage (%) (Sperm Morphology)	Mean Length (Sperm Dimensions)	Mean Width (Sperm Dimensions)
Spermatogonia	Control	30,7	-	-	-
	P1 (0.25 mg/kg BW)	24,8	-	-	-
	P2 (0.5 mg/kg BW)	29,5	-	-	-
	P3 (0.75 mg/kg BW)	25,4	-	-	-
Spermatocytes	Control	26,4	-	-	-
	P1 (0.25 mg/kg BW)	22,8	-	-	-
	P2 (0.5 mg/kg BW)	26,2	-	-	-
	P3 (0.75 mg/kg BW)	23,6	-	-	-
Spermatids	Control	32,9	-	-	-
	P1 (0.25 mg/kg BW)	26,1	-	-	-
	P2 (0.5 mg/kg BW)	31,4	-	-	-
	P3 (0.75 mg/kg BW)	21,3	-	-	-
Overall Spermatogenic Cell Count	Control	29.93 ± 3.37	-	-	-
	P1 (0.25 mg/kg BW)	26.07 ± 4.18	-	-	-
	P2 (0.5 mg/kg BW)	28.54 ± 5.14	-	-	-
	P3 (0.75 mg/kg BW)	23.44 ± 2.26	-	-	-
Normal Spermatozoa	Control	-	88, 88.33 ± 1.97	-	-
	P1 (0.25 mg/kg BW)	-	87, 87 ± 1.79	-	-
	P2 (0.5 mg/kg BW)	-	83, 83.67 ± 2.58	-	-
	P3 (0.75 mg/kg BW)	-	68, 68 ± 4.94	-	-
Abnormal Spermatozoa	Control	-	12, 12 ± 1.9	-	-
	P1 (0.25 mg/kg BW)	-	13, 13 ± 1.79	-	-
	P2 (0.5 mg/kg BW)	-	15, 14.83 ± 1.47	-	-
	P3 (0.75 mg/kg BW)	-	32, 32 ± 4.94	-	-
Sperm Head Dimensions	All Groups (Mean)	-	-	6,21 μm	1,49 μm
Sperm Neck Dimensions	All Groups (Mean)	-	-	9,4 μm	1,31 μm
Sperm Tail Dimensions	All Groups (Mean)	-	-	160 μm	0,877 μm

Data are presented as mean ± SD. Statistical analysis was performed using One-Way ANOVA followed by LSD test (p = 0.043).

Based on the measurements of sperm length and width, the mean head length was 6.21 μm, the neck length was 9.4 μm, and the tail length was 160 μm ( Table 2). The mean width of the spermatozoa was 1.49 μm for the head, 1.31 μm for the neck, and 0.877 μm for the tail. The results showed that the average dimensions of spermatozoa in male rats after administration of *P. indica* stigmasterol differed significantly between the treatment groups compared to the control group. The P3 group, which received the 0.75 mg/kg BW dose, demonstrated the most pronounced reduction in sperm size.

### In Silico target prediction and network analysis

**Figure 3.**
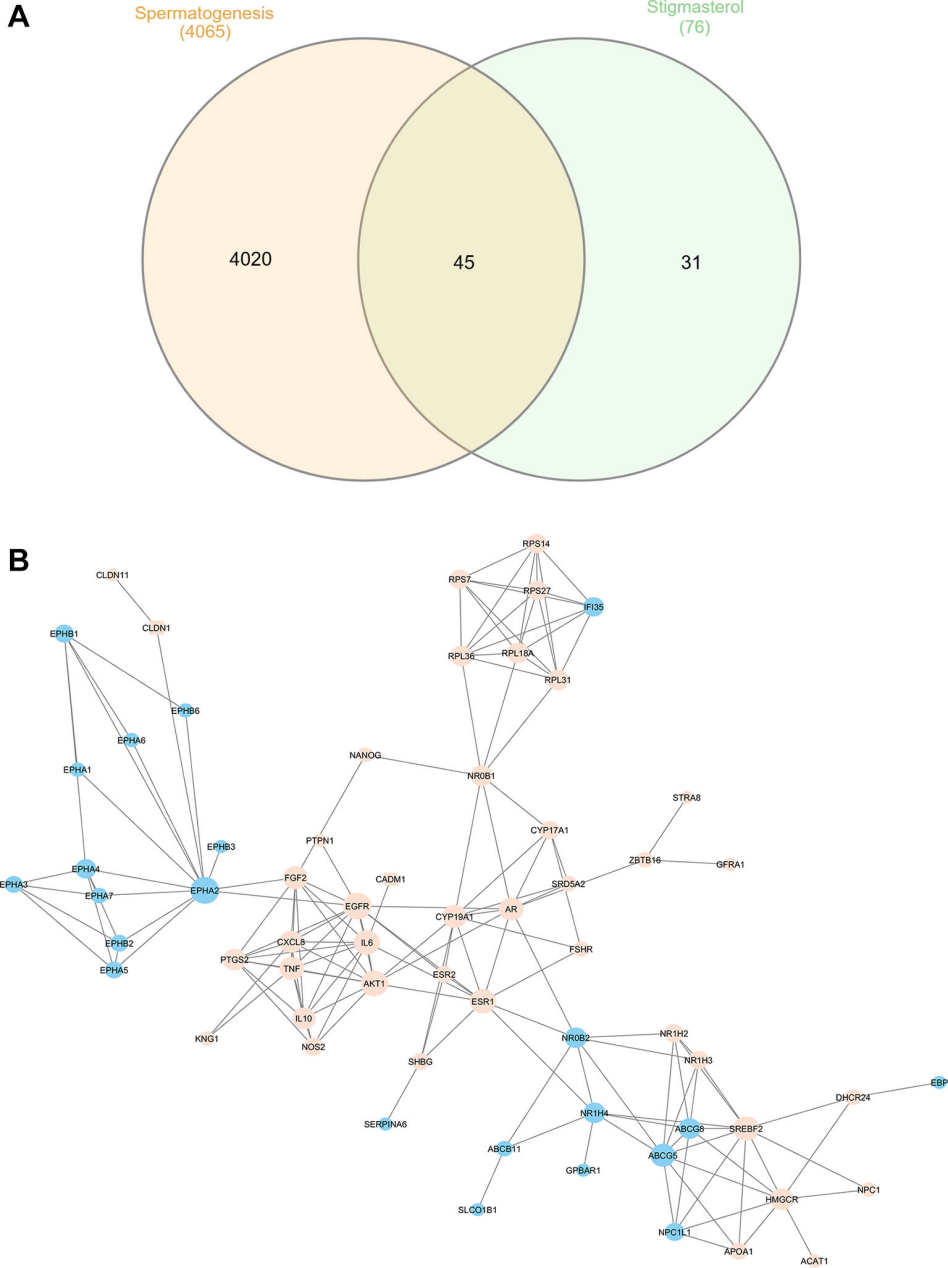
(A) Venn diagram of stigmasterol targets and spermatogenesis-related proteins. (B) Protein-protein interaction network. Blue nodes: stigmasterol targets; orange circles: spermatogenesis-related direct targets; orange squares: indirect targets.

Computational analysis predicted 76 proteins as potential targets of stigmasterol, including AR, FSHR, ESR1, EGFR, and IL6. Among these, 45 proteins were associated with spermatogenesis as seen in Figure 3A. Functional enrichment using DAVID identified pathways related to male gonad development, negative regulation of cell proliferation, and inflammatory responses **(** Table 3). Network analysis revealed that EGFR, EPHA2, AKT1, SREBF2, IL6, and ESR1 had the highest degree and closeness centrality scores, indicating they may play central roles in stigmasterol’s mechanism. Functional enrichment using DAVID identified pathways related to male gonad development, negative regulation of cell proliferation, and inflammatory responses **(** Table 4).

**Table 3.** Functional analysis of stigmasterol targets with p-value (FDR) < 0.05.

ID	Terminology	Count	Genes	FDR
GO:0008584	Male gonad development	6	AR, SRD5A2, FSHR, GFRA1, NR0B1, ESR1	0.0017
GO:0008285	Negative regulation of cell population proliferation	9	IL10, PTPN1, AR, IL6, CXCL8, ZBTB16, DHCR24, IFI35, PTGS2	0.0053
GO:0050728	Negative regulation of inflammatory response	6	IL10, NR1H2, NR1H4, NR1H3, APOA1, RORA	0.0069
GO:0006954	Inflammatory response	8	IL6, CXCL8, NOS2, NR1H4, AKT1, TNF, KNG1, EPHA2	0.0228
GO:0001938	Positive regulation of endothelial cell proliferation	4	IL10, AKT1, FGF2, VEGFA	0.0380

**Table 4.** Top 10 degree centrality dan closeness centrality target stigmasterol.

Name	Degree	Closeness centrality
EGFR	12	0.405
EPHA2	12	0.330
AKT1	11	0.373
SREBF2	10	0.274
IL6	10	0.344
ESR1	10	0.405
ABCG5	9	0.291
FGF2	9	0.342
AR	9	0.408
CYP19A1	9	0.352

## Discussion

This study provides evidence that stigmasterol isolated from *P. indica* leaves significantly reduces spermatogenesis and alters sperm morphology in male rats. The *in vivo* findings demonstrated that administration of stigmasterol for 45 days caused a dose-dependent reduction in spermatogenic cell counts and an increase in sperm morphological abnormalities, particularly at the highest dose tested (0.75 mg/kg BW). This observation aligns with previous research indicating that phytosterols can disrupt hormonal balance and induce germ cell apoptosis. ^37– 40^

This study provides evidence that stigmasterol isolated from *P. indica* leaves significantly reduces spermatogenesis and alters sperm morphology in male rats. The *in vivo* findings demonstrated that administration of stigmasterol for 45 days caused a dose-dependent reduction in spermatogenic cell counts and an increase in sperm morphological abnormalities, particularly at the highest dose tested (0.75 mg/kg BW). This observation aligns with previous research indicating that phytosterols can disrupt hormonal balance and induce germ cell apoptosis. ^41– 43^

Stigmasterol is classified as a phytosterol with structural similarity to testosterone. This similarity may enable stigmasterol to interfere with hormonal feedback mechanisms regulating the hypothalamic-pituitary-gonadal axis, resulting in reduced secretion of luteinizing hormone (LH) and follicle-stimulating hormone (FSH). ^20, 24, 28, 44, 45^ Lower testosterone impairs spermatogenesis by preventing the maturation of spermatogonia and reducing Sertoli cell support. ^46– 49^

Additionally, stigmasterol may increase the conversion of unbound testosterone into dihydrotestosterone (DHT) via 5α-reductase, which binds androgen receptors in Sertoli cells, ultimately inhibiting protein synthesis required for meiosis. Proper Sertoli cell function and hormonal regulation are crucial for maintaining spermatogenesis. ^46– 48^

Morphological assessment revealed a notable increase in abnormal sperm forms across treatment groups. The highest-dose group showed approximately 32% abnormal sperm compared to 12% in the control. Structural abnormalities included detached heads, bent necks, coiled tails, and incomplete segments. These findings align with criteria describing normal sperm morphology as possessing an intact head, neck, and tail. Such defects compromise sperm motility and fertilization potential. ^50– 53^

Testosterone plays a key role in spermiogenesis and maintaining normal sperm structure. A reduction in testosterone, as likely occurred in this study, disrupts cytoplasmic remodeling and differentiation. ^46, 54^ Other factors, including oxidative stress, increased temperature, and epididymal dysfunction, can further impair sperm quality. ^55– 57^ Additionally, genetic and environmental factors contribute to sperm damage and morphological defects. ^58– 60^

Scanning electron microscopy revealed that stigmasterol exposure also led to reductions in sperm size, with the mean head length measuring 6.21 μm and the tail length averaging 160 μm. Such size reduction may impact motility and capacity to fertilize the ovum.

The observed impairments are consistent with prior studies showing stigmasterol’s potential to decrease testosterone, FSH, LH, and overall sperm quality. LH stimulates Leydig cells to produce testosterone, while FSH promotes Sertoli cell activity, androgen-binding protein production, and spermatogonia differentiation. Consequently, disruption of these pathways compromises spermatogenesis and semen characteristics.

The *in silico* network pharmacology analysis provided additional mechanistic insight, identifying ten hub proteins—EGFR, EPHA2, AKT1, ESR1, IL6, AR, TNF, FSHR, SREBF2, and CYP19A1—with high degree and closeness centrality scores. EGFR and AKT1 regulate germ cell proliferation and survival, while ESR1 and AR mediate androgen and estrogen signaling in the testes (Cooke & Walker, 2022). IL6 and TNF are key inflammatory mediators contributing to oxidative stress and germ cell apoptosis. FSHR supports Sertoli cell functions, and CYP19A1 and SREBF2 regulate steroid metabolism and testosterone production. EPHA2 contributes to cell adhesion and germ cell maturation, underscoring the multi-target nature of stigmasterol’s antifertility action. ^61, 62^

Taken together, these findings indicate that stigmasterol acts through multiple interconnected mechanisms, including hormonal disruption, suppression of cell proliferation, and promotion of inflammatory signaling. These pathways are consistent with the in vivo evidence of decreased spermatogenic cell counts, increased sperm abnormalities, and reduced sperm dimensions, supporting the potential development of stigmasterol as a natural male contraceptive agent. However, further studies are needed to assess hormonal parameters, oxidative stress markers, and the reversibility of these effects to confirm the safety and efficacy of stigmasterol for long-term use.

## Conclusion

Stigmasterol from *P. indica* leaves at a dose of 0.75 mg/kg BW significantly reduced spermatogenic cell counts and worsened sperm morphology compared to the other treatment groups. In silico analysis further supported these findings by identifying key protein targets involved in hormonal regulation, germ cell proliferation, and inflammatory pathways that may underlie its antifertility effects.

## Ethical considerations

This study received ethical clearance from the Ethics Committee of the Faculty of Medicine, Universitas Muhammadiyah Malang, Indonesia (No. E54/255/KEPUMM/VIII/2023) on 31 August 2023.

## Data Availability

The underlying data, extended data, ARRIVE checklist, and uncropped/unedited parent images supporting this article are available from Zenodo. DOI: https://doi.org/10.5281/zenodo.19802806, under a License: CC0 1.0 license. ^63^ Not applicable.
