# Stigmasterol from Pluchea indica Leaves Reduces Spermatogenesis and Alters Sperm Morphology in Male Rat: In Vivo and In Silico Study

**DOI:** 10.12688/f1000research.180671.2

**Published:** 2026-06-08

**Authors:** Poncojari Wahyono, Kiky Martha Ariesaka, Eko Susetyarini, H. Husamah

**Affiliations:** 1 Department of Biology Education, Universitas Muhammadiyah Malang, Malang, East Java, Indonesia; 2 Department of Medicine, State University of Malang, Malang, East Java, Indonesia

**Keywords:** Sperm Morphology, Pluchea indica, Rattus norvegicus, Spermatogenesis, Stigmasterol, In Silico Network Pharmacology

## Abstract

**Background:**

Limited male contraception choices have prompted the hunt for natural antifertility drugs. This experimental study investigates the antifertility effects of stigmasterol extracted from *Pluchea indica* leaves in male rats ( *Rattus norvegicus*). Because reproductive hormones were not directly measured, mechanistic interpretations related to testosterone, luteinizing hormone, and follicle-stimulating hormone are presented as possible explanations rather than confirmed endocrine effects.

**Methods:**

This was a randomized experimental study involving twenty-four male rats, which were randomly allocated into four groups (n = 6/group): one control group and three treatment groups receiving stigmasterol at 0.25, 0.5, and 0.75 mg/kg body weight orally for 45 days. Histological examination of testicular tissue and evaluation of sperm morphology were performed to assess spermatogenic activity. Epididymal spermatozoa were collected after euthanasia, diluted, stained with eosin, and evaluated microscopically using standardized morphological criteria. To elucidate possible molecular mechanisms, an in silico network pharmacology analysis was conducted using SEA, CTD, DAVID, STRING, and Cytoscape platforms.

**Results:**

A significant reduction in spermatogenic cell counts and increased sperm morphological abnormalities were observed, particularly at the highest dose (0.75 mg/kg BW) (p = 0.043). Network pharmacology analysis identified 76 predicted protein targets related to spermatogenesis, hormonal regulation, and inflammation, including EGFR, IL6, TNF, ESR1, and AKT1.

**Conclusions:**

This experimental study demonstrates that stigmasterol from *P. indica* leaves, particularly at 0.75 mg/kg BW, exerts antifertility effects by reducing spermatogenic cells and inducing sperm abnormalities in male rats. However, because fertility trials, reversibility assessment, reproductive hormone assays, and comprehensive systemic toxicity evaluation were not conducted, these findings should be interpreted as preliminary preclinical evidence requiring further validation.

## Introduction

The main problem faced in Indonesia in the field of population is the still high rate of population growth. ^1^ According to data from Statistics Indonesia, the population density in Indonesia in 2022 reached 275,773,800 and in 2023 increased to 278,696,200 people. ^2^ Government efforts to control the rising population are carried out through the Family Planning Program regulated by Law No. 52 Article 1 of 2009. The Family Planning Program offers several contraceptive methods. ^3, 4^ Field conditions show that contraception is more directed at women because options for men are still very limited. Therefore, further research is needed to discover safe contraceptive methods for men. One of the approaches being explored is the use of traditional medicinal plants.

Traditional medicines intended for public use must comply with requirements established in distribution permits and must not pose health risks to users. ^5– 11^ Three main aspects need to be considered for any medicinal product to be distributed: safety, quality, and efficacy. ^12– 14^ The Indonesian Food and Drug Authority Regulation Number 10 of 2022 states that to ensure the safety of any medicinal substance, including herbal medicines intended for humans, preclinical in vivo testing must be conducted, including toxicity and efficacy assessments. ^15, 16^

*Pluchea indica* leaves contain many active compounds, such as alkaloids, flavonoids, tannins, essential oils, sodium, potassium, aluminum, calcium, magnesium, phosphorus, saponins, and steroids. Flavonoids, alkaloids, and tannins have been reported to affect spermatogenesis, testosterone levels, and the number of offspring in female white rats. ^17, 18^ Among the compounds with potential antifertility activity is stigmasterol. ^19^ Stigmasterol belongs to the phytosterol group, which is a derivative of steroid compounds. ^20– 24^

Stigmasterol is a phytosterol with steroid-like structural properties and has been reported in previous studies to interact with biological processes related to steroid metabolism, inflammation, oxidative stress, and cell proliferation. ^20^ However, its reproductive effects may vary depending on dose, biological context, and experimental model. Therefore, in this study, stigmasterol was evaluated as a candidate compound that may influence spermatogenic activity and sperm morphology. Because serum testosterone, luteinizing hormone (LH), and follicle-stimulating hormone (FSH) were not directly measured, this study does not claim confirmed endocrine disruption; instead, possible hormonal involvement is discussed as a mechanistic hypothesis supported by previous literature and in silico target prediction. Similar mechanisms of hormonal modulation have been described in other contexts, such as the antiproliferative effects mediated by GnRH receptor signaling pathways. ^25– 28^ Parameters that can be used to assess the quality of the reproductive system include the weight of reproductive organs, sperm abnormalities, sperm count, morphology, and motility. Sperm morphology plays an important role in determining function, especially in the ability to penetrate cervical mucus and interact with the zona pellucida. ^29– 31^ Therefore, sperm shape has an essential role. Previous researches also reported that the assessment of sperm quality includes abnormalities, viability, concentration, and the count of motile spermatozoa. ^24, 32^ Previous studies on male antifertility agents generally focused on these parameters, ^32– 35^ while this study focuses specifically on spermatogenic cell counts and sperm morphology, which remain less explored.

In addition to the in vivo experiments, this research also incorporated in silico network pharmacology analysis to comprehensively predict the molecular targets and biological pathways through which stigmasterol may exert its antifertility effects. This integrative approach is expected to provide mechanistic insights supporting the experimental findings. The selected doses of 0.25, 0.5, and 0.75 mg/kg BW were chosen based on dose ranges used in previous preclinical studies of stigmasterol from *P. indica* leaves and related safety observations in male rats, as well as feasibility for repeated oral administration over one spermatogenic cycle. These doses were intended to explore a low-to-higher dose gradient rather than to establish a definitive therapeutic or contraceptive dose.

## Methods

### Study design

This experimental study was conducted using a completely randomized design (CRD). The treatment period lasted for 45 days. Twenty-four adult male *Rattus norvegicus* aged 5–6 weeks with an average body weight of 200 g were randomly assigned into four groups: one control group receiving distilled water/aquades as the vehicle and three treatment groups receiving stigmasterol isolated from *P. indica* leaves at doses of 0.25 mg/kg BW (P1), 0.5 mg/kg BW (P2), and 0.75 mg/kg BW (P3). Each group consisted of six rats. The study was designed to evaluate the in vivo antifertility effects of stigmasterol from *P. indica* leaves on spermatogenic cells and sperm morphology, supported by network pharmacology analysis.

Each rat was considered an experimental unit. The sample size consisted of six rats per group and 24 rats in total. No formal a priori sample size calculation was conducted; the sample size was determined based on the completely randomized experimental design, previous related preclinical studies, feasibility, and the reduction principle in animal research. Inclusion criteria were healthy and active adult male rats aged 5–6 weeks with an average body weight of approximately 200 g. No animals were excluded from the final analysis. After acclimatisation, rats were randomly allocated into the control and treatment groups. The specific random sequence generation method was not recorded.

### Plant material, extraction, and isolation of stigmasterol

The *P. indica* leaf simplicia used as the source material for stigmasterol isolation was obtained from the Herbal Laboratory of Materia Medika (Lahor Street No.87, Pesanggrahan, Batu City, East Java, Indonesia). Extraction and isolation were performed at the Mark Herb Laboratory, Bandung Institute of Technology, Indonesia (ITB Innovation Park Bandung Technopolis, Bulevar Utama Street No. 3, Cisaranten Kidul, Gedebage, Bandung, West Java, Indonesia).

The dried *P. indica* leaf simplicia was extracted by simple maceration using methanol as the extraction solvent. A total of 500 g of crude dried leaf simplicia was macerated in methanol at a ratio of 1:10 w/v for 72 h at room temperature, with occasional stirring. The macerate was filtered, and the residue was re-macerated twice using fresh methanol to maximize extraction efficiency. The combined filtrates were concentrated under reduced pressure using a rotary evaporator at 45°C. Based on laboratory-scale extraction records commonly used for this type of plant material, the crude methanol extract yield was estimated to be approximately 10–12% of the dried simplicia.

The crude extract was fractionated using an n-hexane–ethyl acetate solvent system and further purified by column chromatography using silica gel 60 as the stationary phase. Elution was performed using an n-hexane:ethyl acetate gradient from non-polar to semi-polar ratios. Fractions with similar thin-layer chromatography profiles were combined. Thin-layer chromatography was performed using silica gel 60 F254 plates and n-hexane:ethyl acetate:formic acid 8:2:0.1 v/v/v as the mobile phase. Spots were visualized under ultraviolet light at 254 and 366 nm and further detected using Liebermann–Burchard reagent. Fractions suspected to contain stigmasterol were purified further and recrystallized using methanol to obtain stigmasterol as a white crystalline powder. The final stigmasterol isolate yield was estimated to be approximately 0.20–0.25% of the crude extract, equivalent to approximately 0.02–0.03% of the dried simplicia.

The identity of stigmasterol was confirmed by thin-layer chromatography comparison with available reference information and its chromatographic behavior as a steroidal compound. No additional high-performance liquid chromatography, liquid chromatography–mass spectrometry, nuclear magnetic resonance spectroscopy, Fourier-transform infrared spectroscopy, or melting point analysis was performed in the present study; therefore, the phytochemical characterization should be interpreted as preliminary and based on chromatographic isolation, thin-layer chromatography confirmation, steroidal reagent detection, and recrystallization. This limitation is acknowledged in the Discussion.

The isolated stigmasterol was used as the test compound and prepared according to the assigned dose for each treatment group. The compound was diluted in distilled water/aquades immediately before oral administration. The plant material was authenticated by Prof. Dr. Elly Purwanti (a professor of botany at Universitas Muhammadiyah Malang, Indonesia). A voucher specimen with the number 303/LAB-BIO/UMM/2025 was deposited at Botany Section of Biology Laboratory, Universitas Muhammadiyah Malang (Raya Tlogomas Street No. 246, Malang, East Java, Indonesia).

### Reagents and materials

All reagents and materials used in the study were documented, including their purpose, amount or concentration, supplier/manufacturer, and catalogue or internal laboratory reference number. The main test compound was stigmasterol isolated from *P. indica* leaves, while distilled water/aquades was used as the vehicle and control treatment. Isoflurane was used as the anesthetic agent, with oxygen as the carrier gas. Histological processing used 10% neutral buffered formalin, graded ethanol, xylene, paraffin wax, hematoxylin, and eosin. Details of the reagents and materials are provided in Table 1.

**Table 1.** Reagents and materials used in the study.

Reagent/material	Purpose	Amount/concentration used	Supplier/manufacturer	Catalogue/internal laboratory reference number
Stigmasterol isolate from *Pluchea indica* leaves	Test compound	0.25, 0.5, and 0.75 mg/kg BW; prepared according to rat body weight	Isolated at Mark Herb Laboratory, Institut Teknologi Bandung, Indonesia	Not applicable; laboratory-isolated compound
*Pluchea indica* leaf simplicia	Source material for stigmasterol isolation	As required for extraction/isolation	Herbal Laboratory of Materia Medika, Batu City, East Java, Indonesia	Not applicable
Distilled water/aquades	Vehicle and control treatment	As required for oral gavage	CV. Krida Tama Persada	88/LB/FK-UMM
Isoflurane	Anesthetic agent	4–5% for induction; 2–3% for maintenance in oxygen	Dexa Medica	201/LB/FK-UMM
Oxygen	Carrier gas for isoflurane anesthesia	As required	Prima Guna Gas	88/LB/FK-UMM
10% neutral buffered formalin	Tissue fixation	10%	Delta Laboratorium	78/LB/FK-UMM
Ethanol	Tissue dehydration	70%, 80%, 90%, 96%, and absolute ethanol	Supelco	112/LB/FK-UMM
Xylene	Tissue clearing	0.03–0.05 mL, 100%	Srikandi Chemical Supplier	152/LB/FK-UMM
Paraffin wax	Tissue embedding	As required	Delta Laboratorium	79/LB/FK-UMM
Hematoxylin	Nuclear staining	1% for 3 minutes	PT Risky Putra Kasih	301/LB/FK-UMM
Eosin	Cytoplasmic staining	1% for 3 minutes	PT Risky Putra Kasih	302/LB/FK-UMM
Oral gavage needle/sonde	Oral administration	As required	Delta Laboratorium	54/LB/FK-UMM

### Preparation and maintenance of experimental animals

Animals were acclimatized for 7 days in plastic cages (45 cm × 30 cm × 15 cm), with each cage housing three rats. The ambient temperature was maintained at 20–25°C. Rats received a daily diet equivalent to 10% of body weight and had free access to distilled water. Bedding material (rice husks) was replaced twice weekly.

Animals were monitored daily for general health, behaviour, food and water intake, and signs of pain or distress, including reduced mobility, abnormal posture, severe lethargy, laboured breathing, marked body weight loss, or persistent refusal to eat or drink. Humane endpoints were established to allow early euthanasia if severe distress or deteriorating health occurred. No unexpected adverse events were observed during the study.

All animal procedures were conducted under controlled laboratory conditions and were designed to minimize pain, distress, and unnecessary suffering. The study protocol was approved by Ethics Committee of the Faculty of Medicine, Universitas Muhammadiyah Malang, Indonesia, under approval number No. E54/255/KEPUMM/VIII/2023 on 31/08/2023. The experimental study commenced on 01/09/2023, after the approval had been granted.

This study did not involve privately owned animals. All animals used in this study were laboratory rats obtained for experimental purposes under the approved institutional animal ethics protocol; therefore, owner informed consent was not applicable.

### Treatment administration

Body weights were measured before treatment initiation and prior to dissection using an analytical balance. The assigned doses of stigmasterol were administered orally using an oral gavage. The control group received distilled water/aquades as the vehicle, while the treatment groups received stigmasterol at the assigned doses. Each dose was prepared according to the body weight of each rat immediately before administration to ensure dosing accuracy.

The assigned dose was adjusted according to the most recent body weight of each rat. Stigmasterol was diluted in distilled water/aquades immediately before administration. The preparation was administered once daily by oral gavage for 45 days. The control group received distilled water/aquades using the same route and schedule.

The doses of 0.25, 0.5, and 0.75 mg/kg BW were selected to represent graded low, intermediate, and higher exposure levels for repeated oral administration. The dose selection was informed by previous preclinical work on stigmasterol from P. indica leaves and related safety observations in male rats, which indicated that these doses were feasible for oral administration without causing observable acute distress during the treatment period. The 45-day administration period was chosen because it covers a substantial portion of the spermatogenic process in rats and allows observation of changes in spermatogenic cells and epididymal sperm morphology. Nevertheless, these doses should not be interpreted as established contraceptive doses, because fertility trials, reversibility testing, and comprehensive toxicity evaluation were not conducted.

### Anesthesia, Euthanasia, and sample collection

After the treatment period, all rats were humanely handled to minimize stress before tissue collection. The animals were anesthetized using isoflurane inhalation. Anesthesia was induced with 4–5% isoflurane in oxygen in an induction chamber and maintained with 2–3% isoflurane in oxygen until a deep anesthetic plane was achieved. The depth of anesthesia was confirmed by the absence of pedal withdrawal and corneal reflexes.

Under deep anesthesia, the animals were euthanized by cervical dislocation as a secondary physical euthanasia method performed by trained personnel. Death was confirmed by the absence of heartbeat, respiration, and reflex responses before dissection. The euthanasia procedure was conducted in accordance with the approved institutional animal ethics protocol and the principles of the American Veterinary Medical Association Guidelines for the Euthanasia of Animals. ^36^

After death was confirmed, testes and epididymides were collected for histological and sperm evaluations. Liver and kidney tissues were also collected as part of the broader sample collection procedure; however, histopathological or biochemical analysis of these organs was not included in the present article because the primary outcomes were spermatogenic cell counts and sperm morphology. Therefore, references to liver and kidney sampling are reported only as sample collection information and are not interpreted as outcome parameters in the Results. Testes were cleaned and fixed in 10% neutral buffered formalin, processed for paraffin embedding, and stained with hematoxylin-eosin for histological observation 16. Sperm samples were diluted with 0.5 mL TRIS buffer and further diluted 200-fold using 0.9% NaCl solution 10. The suspension was homogenized and prepared on glass slides for evaluation.

Epididymal spermatozoa were collected immediately after euthanasia. The cauda epididymis was carefully separated from surrounding fat and connective tissue, placed in a clean Petri dish containing 0.5 mL TRIS buffer, and gently minced using sterile scissors to allow spermatozoa to swim out into the medium. The sperm suspension was gently homogenized and further diluted 200-fold using 0.9% NaCl solution before microscopic evaluation. Fresh preparations were used for morphology assessment to minimize artefactual deformation.

### Observation of spermatogenic cells and sperm morphology

Sperm morphology was assessed by mixing one drop of sperm suspension with 1% eosin, placing the mixture on a clean glass slide, covering it with a coverslip, and examining it under a light microscope at 400× magnification. For each animal, 200 spermatozoa were evaluated from at least five randomly selected microscopic fields. The percentage of normal and abnormal spermatozoa was calculated by dividing the number of spermatozoa in each category by the total number of spermatozoa examined and multiplying the value by 100. Spermatozoa were classified as normal when the head, midpiece/neck, and tail were intact and structurally continuous. Abnormal spermatozoa were identified based on head defects, detached heads, headless tails, bent or wavy necks, coiled tails, broken tails, angled tails, and other incomplete or distorted structures. These criteria were adapted from standard sperm morphology assessment principles for laboratory animals and World Health Organization-based morphology concepts, with adjustment for rat sperm characteristics.

To reduce observational bias, microscopic slides were coded before evaluation, and the observer assessed sperm morphology without knowing the treatment group. The same observer evaluated all slides using identical criteria to maintain intra-observer consistency. Because the slides were not independently evaluated by a second observer, formal inter-observer reliability statistics were not calculated. This limitation is acknowledged in the Discussion.

Spermatogenic cell counts, including spermatogonia, spermatocytes, and spermatids, were determined by observing five randomly selected seminiferous tubule fields per animal under a light microscope at 400× magnification. Cells were identified based on their position within the seminiferous epithelium and their morphological characteristics. Spermatogonia were identified near the basal compartment, spermatocytes were identified as larger cells located more adluminally, and spermatids were identified as smaller post-meiotic cells closer to the tubular lumen. The mean value per animal was used as the experimental unit for statistical analysis.

Microscopic observations were conducted using coded slides where possible to reduce observer bias. Uncropped and unedited parent microscopic images were retained as underlying data. Representative cropped images used in the manuscript were prepared only for presentation purposes, while the original parent images were deposited as underlying data in the repository.

### Network pharmacology analysis

The SMILES structure of stigmasterol (PubChem ID: 5280794) was retrieved from PubChem. Two databases were used to predict target proteins: SEA Target ( https://sea.bkslab.org/) with a p-value cutoff <0.05 and Tanimoto coefficient ≥ 0.4, and the Comparative Toxicogenomics Database (CTD) ( https://ctdbase.org/). Target annotations were performed using the DAVID web server ( https://david.ncifcrf.gov/) for Gene Ontology Biological Process enrichment (p < 0.05). Proteins associated with spermatogenesis were retrieved from GeneCards ( https://www.genecards.org/). Protein–protein interaction networks were analyzed using STRING v12.0 ( https://string-db.org/) with Homo sapiens as the organism and a confidence score ≥ 0.7. The human database was used because human protein annotations and interaction networks are more comprehensively curated in STRING, DAVID, and related databases than rat-specific reproductive annotations. However, because the in vivo experiment used a rat model, the in silico findings were interpreted only as cross-species mechanistic predictions and not as direct evidence of molecular changes in rat testicular tissue. Where relevant, predicted targets were compared conceptually with reproductive processes observed in the rat model.

The TSV output was imported into Cytoscape v3.10 for network topology analysis, including degree and closeness centrality calculations. All databases were accessed on July 4th, 2025. Input files, predicted target lists, enrichment outputs, STRING interaction files, and Cytoscape network files were retained and deposited as underlying or extended data. KEGG pathway enrichment analysis was also performed using DAVID with a significance threshold of p < 0.05 to identify biological pathways associated with the predicted targets. The enriched pathways were used to support interpretation of possible molecular mechanisms but were not considered experimentally validated pathways.

Molecular docking was not performed in the current version of the study. Therefore, the network pharmacology analysis should be interpreted as a target-prediction and pathway-enrichment approach rather than ligand–protein binding validation. This limitation is explicitly stated in the Discussion.

### Data analysis

Data were processed using IBM SPSS Statistics software version 26.0. The animal was used as the experimental unit, and each group consisted of six rats. Data were first examined for normality using the Shapiro–Wilk test and for homogeneity of variance using Levene’s test. When assumptions of normality and homogeneity were met, one-way analysis of variance was used to determine differences among groups. When the analysis of variance result was significant, post hoc comparisons were performed using the Least Significant Difference test. The level of significance was set at p ≤ 0.05.

Statistical results are reported using mean ± standard deviation, F values, degrees of freedom, exact p-values, and eta squared values (η ^2^) as effect size estimates where applicable. A 95% confidence interval was added to support interpretation of the magnitude and precision of treatment effects. Exact statistical outputs, including normality, homogeneity, analysis of variance, post hoc tests, and animal-level data, were prepared for deposition as underlying data.

The exact number of animals included in each analysis was six per group. No animals or data points were excluded from the final analysis. Individual animal-level data, values underlying means and standard deviations, values used to generate figures, and statistical output files were prepared for deposition in an open repository.

## Results

### Spermatogenic cell counts

The administration of stigmasterol from *P. indica* leaves affected spermatogenic cell numbers in male rats ( *Rattus norvegicus*). Table 1 shows the mean counts of spermatogonia, spermatocytes, and spermatids across treatment groups. The control group exhibited higher average counts of spermatogenic cells compared to all treatment groups. In particular, the highest stigmasterol dose (0.75 mg/kg BW) resulted in the lowest counts of spermatogonia (24.4), spermatocytes (23.6), and spermatids (21.3).

**Figure 1.**
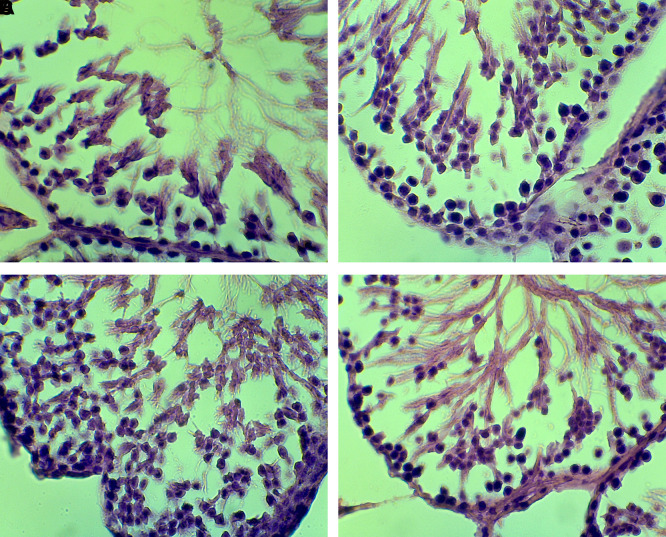
(A) Histology of rat testes stained with hematoxylin-eosin (400× magnification); K: control. (B) Histology of rat testes stained with hematoxylin-eosin (400× magnification); P1: treatment 1. (C) Histology of rat testes stained with hematoxylin-eosin (400× magnification); P2: treatment 2. (D) Histology of rat testes stained with hematoxylin-eosin (400× magnification); P3: treatment 3.

Statistical analysis using one-way ANOVA revealed a significant difference in overall spermatogenic cell counts among groups, F(3,20) = 3.25, exact p = 0.0435, η ^2^ = 0.328, indicating a moderate-to-large treatment effect. The 95% confidence intervals were 26.39–33.47 for the control group, 21.68–30.46 for P1, 23.15–33.93 for P2, and 21.07–25.81 for P3. The LSD post hoc test indicated that the highest-dose group, P3 (0.75 mg/kg BW), had a significantly lower overall spermatogenic cell count (23.44 ± 2.26) than the control group (29.93 ± 3.37). P1 and P2 showed intermediate values, indicating a tendency toward reduced spermatogenic cell counts after stigmasterol administration. However, the response was not strictly linear across all cell types, because P2 showed higher values than P1 for several spermatogenic cell categories. Therefore, the findings are interpreted as a treatment-associated reduction that was most evident at 0.75 mg/kg BW, rather than as a fully linear dose-response pattern across all doses ( Table 1). Representative histological images are presented in Figure 1 **(A-D)**.

Liver and kidney samples were collected during necropsy but were not analyzed as outcome variables in the present article. Therefore, no liver or kidney histological or biochemical results are presented. The absence of these data is acknowledged as a limitation, and future studies should include systemic toxicity evaluation when assessing the safety profile of stigmasterol.

### Sperm morphologic

**Figure 2.**
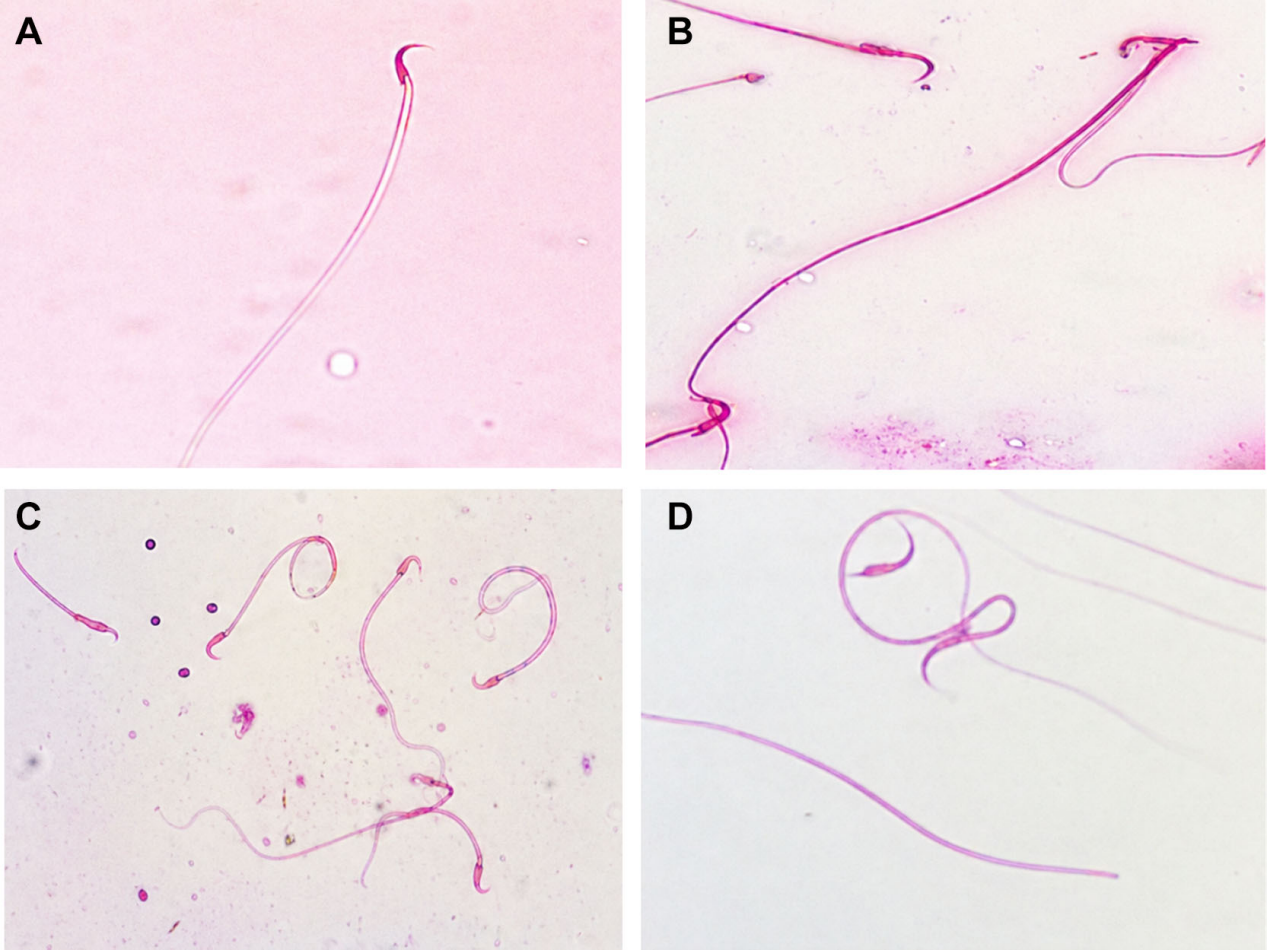
(A) Rat spermatozoa: K = Control group. (B) Rat spermatozoa: P1 = Treatment 1. (C) Rat spermatozoa: P2 = Treatment 2. (D) Rat spermatozoa: P3 = Treatment 3.

Observations presented in Figure 2 **(A-D)** show that white rats possess spermatozoa with normal morphology, consisting of a head, neck, and tail. However, in abnormal spermatozoa, these parts were incomplete and exhibited various structural defects. In the control group, spermatozoa appeared normal, with intact components including the head, neck, and tail. In contrast, the treatment groups showed multiple abnormalities, such as heads lacking an acrosome, headless tails, detached heads, wavy necks, coiled tails, tails forming an angle, and tails without a head.

The highest mean percentage of sperm abnormalities was observed in the P3 group (0.75 mg/kg BW) at 32%. In contrast, the control group showed the lowest mean abnormality percentage, with only 12%. Regarding the percentage of normal spermatozoa, the highest mean was found in the control group. Normality and homogeneity tests, followed by One-Way ANOVA and Least Significant Difference (LSD) tests, were performed on the data. The one-way ANOVA test indicated a significant difference among treatment groups for the percentage of both normal and abnormal spermatozoa. Based on the reported mean ± standard deviation values and six animals per group, the estimated ANOVA result for normal spermatozoa was F(3,20) = 55.29, p < 0.001, η ^2^ = 0.892, indicating a very large treatment effect. The 95% confidence intervals for normal spermatozoa were 86.26–90.40% for the control group, 85.12–88.88% for P1, 80.96–86.38% for P2, and 62.82–73.18% for P3. For abnormal spermatozoa, the estimated ANOVA result was F(3,20) = 64.00, p < 0.001, η ^2^ = 0.906, also indicating a very large treatment effect. The 95% confidence intervals for abnormal spermatozoa were 10.01–13.99% for the control group, 11.12–14.88% for P1, 13.29–16.37% for P2, and 26.82–37.18% for P3. Because p-values should not be reported as 0.000, very small p-values displayed by IBM SPSS Statistics as 0.000 are reported as p < 0.001.

As shown in Table 2, significant differences were found among treatments in both normal and abnormal spermatozoa percentages. In the normal spermatozoa category, P3 (68.00 ± 4.94%) showed the lowest percentage compared with the control (88.33 ± 1.97%), P1 (87.00 ± 1.79%), and P2 (83.67 ± 2.58%). Conversely, abnormal spermatozoa were highest in P3 (32.00 ± 4.94%) compared with the control (12.00 ± 1.90%), P1 (13.00 ± 1.79%), and P2 (14.83 ± 1.47%). These findings indicate that the highest dose of stigmasterol was associated with a marked shift from normal to abnormal sperm morphology.

**Table 2.** Effects of Stigmasterol from *Pluchea indica* Leaves on Spermatogenic Cells, Sperm Morphology, and Sperm Dimensions in Male Rats.

Parameter	Group (Dose)	Mean ± SD (Spermatogenic Cell Counts)	Mean Percentage (%) (Sperm Morphology)	Mean Length (Sperm Dimensions)	Mean Width (Sperm Dimensions)
Spermatogonia	Control	30,7	-	-	-
	P1 (0.25 mg/kg BW)	24,8	-	-	-
	P2 (0.5 mg/kg BW)	29,5	-	-	-
	P3 (0.75 mg/kg BW)	25,4	-	-	-
Spermatocytes	Control	26,4	-	-	-
	P1 (0.25 mg/kg BW)	22,8	-	-	-
	P2 (0.5 mg/kg BW)	26,2	-	-	-
	P3 (0.75 mg/kg BW)	23,6	-	-	-
Spermatids	Control	32,9	-	-	-
	P1 (0.25 mg/kg BW)	26,1	-	-	-
	P2 (0.5 mg/kg BW)	31,4	-	-	-
	P3 (0.75 mg/kg BW)	21,3	-	-	-
Overall Spermatogenic Cell Count	Control	29.93 ± 3.37	-	-	-
	P1 (0.25 mg/kg BW)	26.07 ± 4.18	-	-	-
	P2 (0.5 mg/kg BW)	28.54 ± 5.14	-	-	-
	P3 (0.75 mg/kg BW)	23.44 ± 2.26	-	-	-
Normal Spermatozoa	Control	-	88, 88.33 ± 1.97	-	-
	P1 (0.25 mg/kg BW)	-	87, 87 ± 1.79	-	-
	P2 (0.5 mg/kg BW)	-	83, 83.67 ± 2.58	-	-
	P3 (0.75 mg/kg BW)	-	68, 68 ± 4.94	-	-
Abnormal Spermatozoa	Control	-	12, 12 ± 1.9	-	-
	P1 (0.25 mg/kg BW)	-	13, 13 ± 1.79	-	-
	P2 (0.5 mg/kg BW)	-	15, 14.83 ± 1.47	-	-
	P3 (0.75 mg/kg BW)	-	32, 32 ± 4.94	-	-
Sperm Head Dimensions	All Groups (Mean)	-	-	6,21 μm	1,49 μm
Sperm Neck Dimensions	All Groups (Mean)	-	-	9,4 μm	1,31 μm
Sperm Tail Dimensions	All Groups (Mean)	-	-	160 μm	0,877 μm

Data are presented as mean ± SD. Statistical analysis was performed using One-Way ANOVA followed by LSD test (p = 0.043).

Based on the measurements of sperm length and width, the mean head length was 6.21 μm, the neck length was 9.4 μm, and the tail length was 160 μm ( Table 2). The mean width of the spermatozoa was 1.49 μm for the head, 1.31 μm for the neck, and 0.877 μm for the tail. The results showed that the average dimensions of spermatozoa in male rats after administration of *P. indica* stigmasterol differed significantly between the treatment groups compared to the control group. The P3 group, which received the 0.75 mg/kg BW dose, demonstrated the most pronounced reduction in sperm size.

### In silico target prediction and network analysis

**Figure 3.**
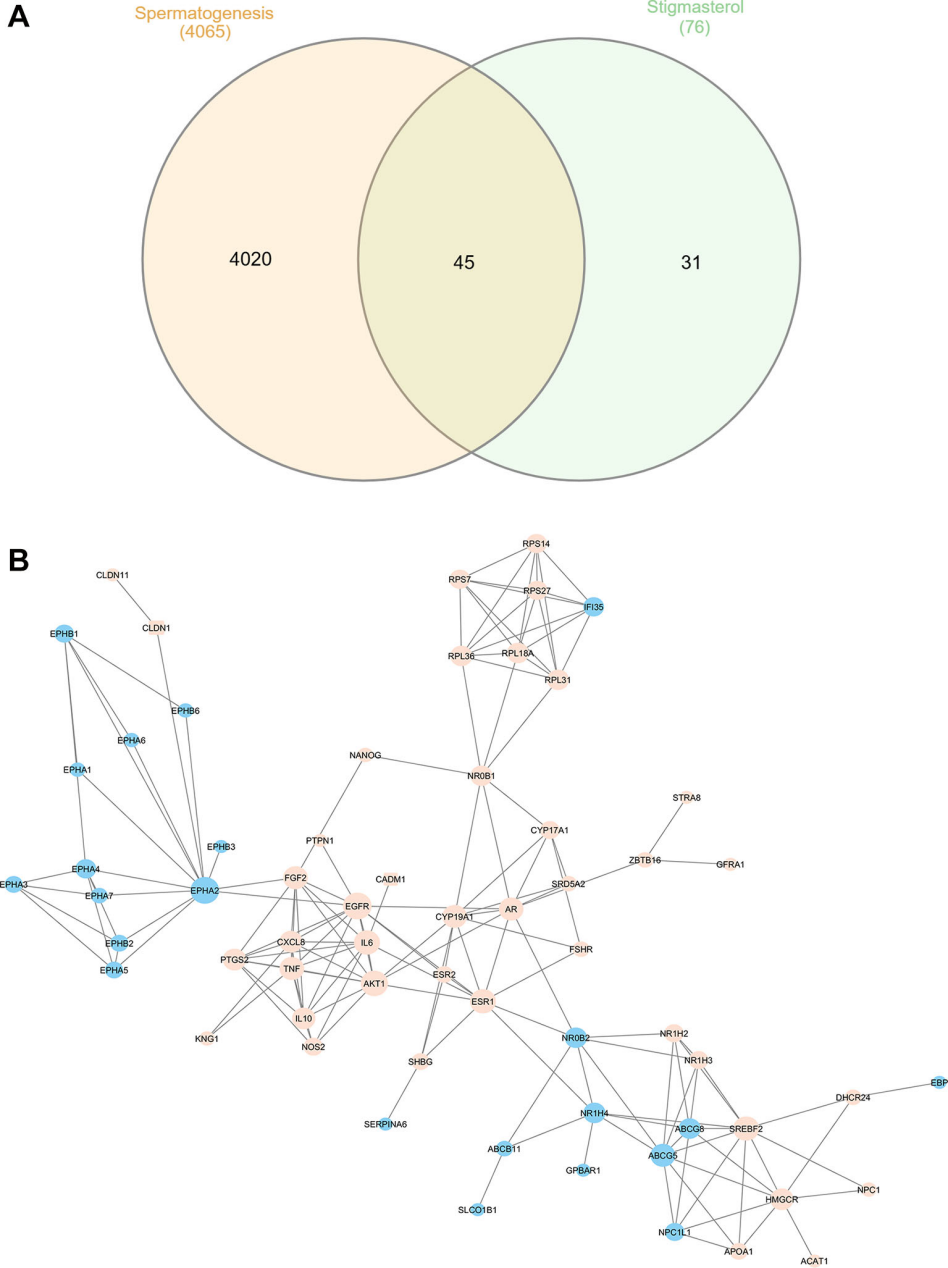
(A) Venn diagram of stigmasterol targets and spermatogenesis-related proteins. (B) Protein-protein interaction network. Blue nodes: stigmasterol targets; orange circles: spermatogenesis-related direct targets; orange squares: indirect targets.

Computational analysis predicted 76 proteins as potential targets of stigmasterol, including AR, FSHR, ESR1, EGFR, AKT1, TNF, and IL6. Among these, 45 proteins overlapped with spermatogenesis-related proteins based on database annotation, as seen in Figure 3A. Functional enrichment using DAVID identified biological processes related to male gonad development, negative regulation of cell population proliferation, inflammatory response, and regulation of inflammatory response **(** Table 3). Network analysis revealed that EGFR, EPHA2, AKT1, SREBF2, IL6, and ESR1 had the highest degree and closeness centrality scores, indicating they may play central roles in stigmasterol’s mechanism. Functional enrichment using DAVID identified pathways related to male gonad development, negative regulation of cell proliferation, and inflammatory responses **(** Table 4).

**Table 3.** Functional analysis of stigmasterol targets with p-value (FDR) < 0.05.

ID	Terminology	Count	Genes	FDR
GO:0008584	Male gonad development	6	AR, SRD5A2, FSHR, GFRA1, NR0B1, ESR1	0.0017
GO:0008285	Negative regulation of cell population proliferation	9	IL10, PTPN1, AR, IL6, CXCL8, ZBTB16, DHCR24, IFI35, PTGS2	0.0053
GO:0050728	Negative regulation of inflammatory response	6	IL10, NR1H2, NR1H4, NR1H3, APOA1, RORA	0.0069
GO:0006954	Inflammatory response	8	IL6, CXCL8, NOS2, NR1H4, AKT1, TNF, KNG1, EPHA2	0.0228
GO:0001938	Positive regulation of endothelial cell proliferation	4	IL10, AKT1, FGF2, VEGFA	0.0380

**Table 4.** Top 10 degree centrality dan closeness centrality target stigmasterol.

Name	Degree	Closeness centrality
EGFR	12	0.405
EPHA2	12	0.330
AKT1	11	0.373
SREBF2	10	0.274
IL6	10	0.344
ESR1	10	0.405
ABCG5	9	0.291
FGF2	9	0.342
AR	9	0.408
CYP19A1	9	0.352

Because the STRING and DAVID analyses were performed using human protein annotations, these findings should be interpreted as predictive and hypothesis-generating. They suggest possible molecular networks that may be relevant to stigmasterol exposure, but they do not prove that the same molecular changes occurred in rat testicular tissue. Molecular docking, reproductive transcriptomic comparison, and experimental validation of target proteins were not performed in the present study.

## Discussion

This study provides experimental evidence that stigmasterol isolated from *P. indica* leaves reduces spermatogenic cell counts and increases sperm morphological abnormalities in male rats. The most prominent changes were observed in the P3 group receiving 0.75 mg/kg BW for 45 days. The control group showed higher spermatogenic cell counts and a higher percentage of normal spermatozoa, whereas the highest-dose group showed the lowest overall spermatogenic cell count and the highest percentage of abnormal spermatozoa. These findings indicate that repeated oral exposure to stigmasterol at the tested doses, particularly 0.75 mg/kg BW, was associated with impaired spermatogenic activity and altered epididymal sperm morphology. ^37– 43^

Stigmasterol is classified as a phytosterol with structural similarity to steroid compounds. This structural feature may allow stigmasterol or related phytosterols to interact with biological pathways associated with steroid metabolism, androgen signaling, and reproductive regulation. However, serum testosterone, LH, and FSH were not measured in this study. Therefore, the present findings cannot directly demonstrate that stigmasterol reduced reproductive hormone levels. ^20, 24, 28, 44, 45^ Instead, hormonal involvement should be interpreted as a plausible mechanism based on previous studies and supported indirectly by the predicted involvement of targets such as AR, FSHR, ESR1, CYP19A1, and SREBF2 in the in silico analysis. ^46– 49^

Previous studies have shown that testosterone, luteinizing hormone, and follicle-stimulating hormone are important regulators of spermatogenesis and Sertoli–Leydig cell function. ^50, 51^ Therefore, a reduction in testosterone or disruption of gonadotropin signaling, if present, could plausibly impair spermatogonial maturation, Sertoli cell support, and spermiogenesis. ^46, 52, 53^ However, because these reproductive hormones were not directly measured in the present study, this mechanism remains hypothetical. Future studies should directly measure serum testosterone, luteinizing hormone, follicle-stimulating hormone, inhibin B, and intratesticular testosterone to verify whether endocrine disruption contributes to the reproductive effects observed in this model.

Similarly, the possibility that stigmasterol may influence 5α-reductase activity, dihydrotestosterone metabolism, or androgen receptor signaling remains hypothetical in the present study. These mechanisms are biologically plausible because androgen signaling is essential for spermatogenesis and Sertoli cell function, but they require direct validation using hormonal assays, enzyme activity tests, or gene/protein expression analysis in testicular tissue. ^46– 48^

Morphological assessment revealed a notable increase in abnormal sperm forms across treatment groups, particularly in P3. The highest-dose group showed approximately 32% abnormal sperm compared to 12% in the control. Structural abnormalities included detached heads, headless tails, bent or wavy necks, coiled tails, angled tails, and incomplete segments. These defects may compromise sperm motility, capacitation, and fertilization capacity, although these functional outcomes were not directly tested in the present study. Therefore, the observed morphological alterations should be interpreted as indicators of impaired sperm quality rather than direct evidence of reduced fertility. ^54– 57^

Testosterone and gonadotropins play key roles in spermiogenesis and the maintenance of normal sperm structure. However, because hormone levels were not assessed, this study cannot determine whether the observed sperm abnormalities were caused by endocrine disruption, oxidative stress, epididymal dysfunction, germ cell damage, or a combination of these mechanisms. ^46, 58^ Other factors, including oxidative stress, increased temperature, and epididymal dysfunction, can further impair sperm quality. ^59, 60^ The consequences of climate change and male reproductive health: A review of the possible impact and mechanisms. ^59– 61^ Additionally, genetic and environmental factors contribute to sperm damage and morphological defects. ^62– 64^ Future research should include reproductive hormone measurement, oxidative stress markers, testicular apoptosis markers, epididymal function assessment, and fertility testing to clarify the causal pathway.

Microscopic measurement of sperm dimensions indicated mean values of 6.21 μm for head length, 9.4 μm for neck length, and 160 μm for tail length. However, because sperm dimensions were summarized across groups and detailed group-level statistical values were limited, these data should be interpreted cautiously. If sperm size is retained as an outcome, future versions should present complete group-specific means, standard deviations, statistical comparisons, and representative measurement images. Otherwise, sperm dimension data should be described as supportive descriptive information rather than a primary outcome.

The observed impairments are consistent with prior studies showing stigmasterol’s potential to decrease testosterone, FSH, LH, and overall sperm quality. LH stimulates Leydig cells to produce testosterone, while FSH promotes Sertoli cell activity, androgen-binding protein production, and spermatogonia differentiation. Consequently, disruption of these pathways compromises spermatogenesis and semen characteristics.

The in silico network pharmacology analysis provided hypothesis-generating insight into possible molecular pathways associated with stigmasterol. The analysis identified several hub proteins, including EGFR, EPHA2, AKT1, ESR1, IL6, AR, TNF, FSHR, SREBF2, and CYP19A1. These proteins are associated with processes such as cell proliferation, inflammatory signaling, steroid metabolism, and reproductive regulation. Nevertheless, the network pharmacology results should not be interpreted as experimentally validated mechanisms. The analysis used human protein databases because these resources provide more complete annotation and interaction data than rat-specific databases. Consequently, cross-species interpretation is required, and the predicted targets need validation in rat testicular tissue through molecular docking, qPCR, Western blotting, immunohistochemistry, or reproductive transcriptomic comparison. ^65– 69^

Taken together, these findings indicate that stigmasterol exposure was associated with reduced spermatogenic cell counts and increased sperm abnormalities in male rats. The in silico analysis further generated plausible molecular hypotheses involving steroid signaling, cell proliferation, and inflammatory pathways. However, the present study does not provide sufficient evidence to support direct contraceptive development claims. Further studies involving fertility trials, mating success assessment, reversibility testing, reproductive hormone assays, systemic toxicity evaluation, and stronger phytochemical characterization are required before stigmasterol can be considered a validated male contraceptive candidate.

This study has several limitations. First, serum testosterone, LH, and FSH were not measured; therefore, endocrine mechanisms remain hypothetical. Second, phytochemical characterization of the stigmasterol isolate was limited to the available extraction, chromatographic isolation, TLC confirmation, and recrystallization procedures; additional HPLC, LC-MS, NMR, FTIR, melting point, and purity analyses would strengthen compound verification. Third, sperm morphology was evaluated microscopically, but future studies should include larger standardized sperm counts per animal, stronger observer-blinding procedures, and intra- or inter-observer reliability testing. Fourth, liver and kidney samples were collected but not analyzed as outcome variables in this article, limiting systemic safety interpretation. Fifth, the in silico analysis was based on predictive human protein databases and did not include molecular docking or experimental validation. Finally, fertility trials, mating success, reversibility, libido, reproductive organ weights, and offspring outcomes were not assessed. These limitations mean that the present findings should be viewed as preliminary preclinical evidence rather than proof of contraceptive efficacy.

## Conclusion

Stigmasterol from *P. indica* leaves, particularly at a dose of 0.75 mg/kg BW, significantly reduced spermatogenic cell counts and increased sperm morphological abnormalities in male rats after 45 days of oral administration. The in silico analysis identified predicted targets and pathways related to steroid signaling, cell proliferation, inflammatory response, and male gonad development, which may help explain the observed reproductive changes. However, because reproductive hormones, fertility outcomes, reversibility, molecular target validation, and comprehensive systemic toxicity were not assessed, these findings should be interpreted as preliminary evidence of reproductive effects rather than definitive proof of male contraceptive efficacy. Further studies integrating hormonal assays, fertility trials, reversibility assessment, liver and kidney safety evaluation, and stronger phytochemical characterization are required.

## Ethical considerations

This study received ethical clearance from the Ethics Committee of the Faculty of Medicine, Universitas Muhammadiyah Malang, Indonesia (No. E54/255/KEPUMM/VIII/2023) on 31 August 2023.

## Data Availability

The underlying data, extended data, ARRIVE checklist, and uncropped/unedited parent images supporting this article are available from Zenodo. DOI: https://doi.org/10.5281/zenodo.19802806, under a License: CC0 1.0 license. ^70^ Not applicable.
