# Supplementary material for: Stigmasterol from Pluchea indica Leaves Reduces Spermatogenesis and Alters Sperm Morphology in Male Rat: In Vivo and In Silico Study
Source: F1000Res. 2026 Jul 9;15:735. Originally published 2026 May 14. [Version 3] doi: 10.12688/f1000research.180671.3 (PMC13250513; doi:10.12688/f1000research.180671.3)
Supplement: Peer review history

# Reviewer report: Reviewer response for version 3

**DOI:** 10.5256/f1000research.205360.r499880

**Published:** 2026-07-11

**Authors:** Ruqiah Ganda Putri Panjaitan

**Affiliations:** 1 Tanjungpura University, Pontianak, Indonesia

This manuscript has been presented well and completely, worthy of indexation.

Is the work clearly and accurately presented and does it cite the current literature?

Yes

If applicable, is the statistical analysis and its interpretation appropriate?

Yes

Are all the source data underlying the results available to ensure full reproducibility?

Yes

Is the study design appropriate and is the work technically sound?

Yes

Are the conclusions drawn adequately supported by the results?

Yes

Are sufficient details of methods and analysis provided to allow replication by others?

Yes

Reviewer Expertise:

Biology, Medicinal Plants, Ethomedicine, Toxicology, and Helath

I confirm that I have read this submission and believe that I have an appropriate level of expertise to confirm that it is of an acceptable scientific standard.

# Reviewer report: Reviewer response for version 2

**DOI:** 10.5256/f1000research.202716.r491838

**Published:** 2026-06-09

**Authors:** Supiana Dian Nurtjahyani

**Affiliations:** 1 Universitas PGRI Ronggolawe Tuban, Tuban, East Java, Indonesia

The authors have addressed all the points raised in my previous review thoroughly and effectively. The revised manuscript (version 2) shows significant improvement in clarity, methodology, and discussion of the results. I appreciate the detailed responses from the authors and the revisions made accordingly. Therefore, I have no further comments or requests for revision. My decision is **APPROVED**.

Is the work clearly and accurately presented and does it cite the current literature?

Yes

If applicable, is the statistical analysis and its interpretation appropriate?

Yes

Are all the source data underlying the results available to ensure full reproducibility?

Yes

Is the study design appropriate and is the work technically sound?

Yes

Are the conclusions drawn adequately supported by the results?

Yes

Are sufficient details of methods and analysis provided to allow replication by others?

Yes

Reviewer Expertise:

Molecular Biology and PCR Diagnostics, Microbiology, Biotechnology and biology education, and Parasitology and Health Biology

I confirm that I have read this submission and believe that I have an appropriate level of expertise to confirm that it is of an acceptable scientific standard.

# Reviewer report: Reviewer response for version 1

**DOI:** 10.5256/f1000research.199297.r486217

**Published:** 2026-06-02

**Authors:** Supiana Dian Nurtjahyani

**Affiliations:** 1 Universitas PGRI Ronggolawe Tuban, Tuban, East Java, Indonesia

1. Figure 1 is marked and captioned and the image resolution is increased.

 2. Figure 2 and Figure 3 image resolution increased.

 3. Table 2 is empty and has an explanation added in the discussion.

 4. The abstract and research objectives need to be made clearer.

 5. Information regarding dosage should be accompanied by references.

Is the work clearly and accurately presented and does it cite the current literature?

Yes

If applicable, is the statistical analysis and its interpretation appropriate?

Yes

Are all the source data underlying the results available to ensure full reproducibility?

Yes

Is the study design appropriate and is the work technically sound?

Yes

Are the conclusions drawn adequately supported by the results?

Yes

Are sufficient details of methods and analysis provided to allow replication by others?

Yes

Reviewer Expertise:

Molecular Biology and PCR Diagnostics, Microbiology, Biotechnology and biology education, and Parasitology and Health Biology

I confirm that I have read this submission and believe that I have an appropriate level of expertise to confirm that it is of an acceptable scientific standard, however I have significant reservations, as outlined above.

# Reviewer report: Reviewer response for version 1

**DOI:** 10.5256/f1000research.199297.r486224

**Published:** 2026-05-27

**Authors:** Ruqiah Ganda Putri Panjaitan

**Affiliations:** 1 Tanjungpura University, Pontianak, Indonesia

In general, the presentation in the Introduction is well-organized and supported by good references. The presentation of the methods is also quite good, but the solvents used for the extraction and fractionation processes should be presented. It is recommended to present the complete amount of crude drugs and the yield of the extraction and fractionation results. The sperm collection procedure should be presented. It is recommended to complete the data analysis, both qualitative and quantitative. The results and discussion sections should be aligned with the parameters measured (adjusted to what is stated in the Methods). For example, in the Methods section, liver and kidney sampling are mentioned, but the results from these organ sampling are not yet presented. Before indexing, it is recommended that revisions be made as suggested.

Is the work clearly and accurately presented and does it cite the current literature?

Yes

If applicable, is the statistical analysis and its interpretation appropriate?

Yes

Are all the source data underlying the results available to ensure full reproducibility?

Yes

Is the study design appropriate and is the work technically sound?

Yes

Are the conclusions drawn adequately supported by the results?

Yes

Are sufficient details of methods and analysis provided to allow replication by others?

Yes

Reviewer Expertise:

Biology, Medicinal Plants, Ethomedicine, Toxicology, and Helath

I confirm that I have read this submission and believe that I have an appropriate level of expertise to confirm that it is of an acceptable scientific standard, however I have significant reservations, as outlined above.

# Response

**Published:** 2026-05-28

**Authors:** Poncojari Wahyono

**Affiliations:** Biology Education, Universitas Muhammadiyah Malang, Malang, East Java, Indonesia

**Response to Reviewers**

 Dear Reviewer,

 We would like to sincerely thank the reviewers for their careful evaluation, constructive comments, and valuable recommendations on our manuscript entitled “Stigmasterol from Pluchea indica Leaves Reduces Spermatogenesis and Alters Sperm Morphology in Male Rat: In Vivo and In Silico Study.” We have revised the manuscript thoroughly according to the reviewers’ suggestions.

 All changes in the revised manuscript have been marked in bold, while deleted or replaced text has been indicated using deletion marks where appropriate. The revisions mainly include clarification of the extraction and isolation procedure, addition of solvent and yield information, explanation of sperm collection and sperm morphology assessment procedures, improvement of statistical reporting, more cautious interpretation of hormonal mechanisms, clarification of the in silico analysis, limitation of contraceptive claims, and addition of a study limitations section.

 Below, we provide a point-by-point response to each reviewer’s comments.

 Response to Reviewer 2

 General comment

**Reviewer comment:**

*In general, the presentation in the Introduction is well-organized and supported by good references. The presentation of the methods is also quite good, but the solvents used for the extraction and fractionation processes should be presented. It is recommended to present the complete amount of crude drugs and the yield of the extraction and fractionation results. The sperm collection procedure should be presented. It is recommended to complete the data analysis, both qualitative and quantitative. The results and discussion sections should be aligned with the parameters measured. For example, in the Methods section, liver and kidney sampling are mentioned, but the results from these organ sampling are not yet presented. Before indexing, it is recommended that revisions be made as suggested.*

**Response:**

 We sincerely thank the reviewer for the positive assessment of the Introduction and Methods sections, as well as for the constructive recommendations to improve the clarity, reproducibility, and alignment of the manuscript. We have carefully revised the manuscript according to all suggestions.

 First, we revised the subsection “Plant material, extraction, and isolation of stigmasterol” by adding details on the extraction solvent, amount of dried Pluchea indica leaf simplicia, solvent ratio, maceration duration, re-maceration process, rotary evaporation temperature, crude extract yield, fractionation solvent system, column chromatography stationary phase, eluent gradient, thin-layer chromatography conditions, visualization method, recrystallization solvent, and estimated stigmasterol isolate yield.

 Second, we added a clearer sperm collection procedure. The revised manuscript now explains that epididymal spermatozoa were collected from the cauda epididymis after euthanasia, diluted in TRIS buffer, further diluted using 0.9% sodium chloride solution, homogenized, stained with eosin, and evaluated microscopically.

 Third, we expanded the sperm morphology methodology by specifying that 200 spermatozoa were evaluated per animal from randomly selected microscopic fields. We also clarified the criteria for normal and abnormal spermatozoa, including head defects, detached heads, headless tails, bent or wavy necks, coiled tails, broken tails, angled tails, and incomplete structures. We further explained the use of coded slides to reduce observational bias.

 Fourth, we revised the Data analysis section to provide a more complete statistical workflow. We clarified that the animal was used as the experimental unit, with six rats per group. We added information on Shapiro–Wilk normality testing, Levene’s homogeneity testing, one-way analysis of variance, Least Significant Difference post hoc testing, exact p-value reporting, eta squared effect size, and 95% confidence intervals.

 Fifth, we aligned the Results and Discussion sections with the parameters actually measured in the study. We clarified that liver and kidney tissues were collected during necropsy but were not analyzed as outcome parameters in the present article. Therefore, no liver or kidney results are presented. This point has also been acknowledged as a limitation of the study.

**Changes made in the manuscript:**

- Methods: “Plant material, extraction, and isolation of stigmasterol”.
- Methods: “Anesthesia, euthanasia, and sample collection”.
- Methods: “Observation of spermatogenic cells and sperm morphology”.
- Methods: “Data analysis”.
- Results: “Spermatogenic cell counts” and “Sperm morphology”.
- Discussion: revised interpretation and added study limitations.

Closing Statement

 We sincerely appreciate the reviewers’ constructive comments, which have helped us improve the clarity, methodological transparency, statistical reporting, and scientific interpretation of the manuscript. We believe that the revised version is now more cautious, better aligned with the measured parameters, and more transparent regarding the limitations of the study.

 Thank you for your valuable time and consideration.

 Sincerely,

 The Authors

# Reviewer report: Reviewer response for version 1

**DOI:** 10.5256/f1000research.199297.r486223

**Published:** 2026-05-26

**Authors:** Anita Restu Puji Raharjeng

**Affiliations:** 1 Universitas Islam Negeri Raden Fatah Palembang, Palembang, South Sumatra, Indonesia

1. Lack of Hormonal Measurements

 The authors repeatedly conclude that stigmasterol disrupts hormonal regulation and reduces testosterone, LH, and FSH. However, no hormonal assays were conducted.

 This is a major weakness because the mechanistic conclusions are speculative.

 Recommendation: The authors should either measure serum testosterone, LH, and FSH levels, or substantially tone down mechanistic claims. Statements such as:

 “Lower testosterone impairs spermatogenesis…”

 should be framed as hypotheses rather than established findings in this study.

 2. Weak Characterization of Stigmasterol Isolation

 The extraction and isolation procedure is inadequately described. The manuscript only mentions chromatography, TLC confirmation, and recrystallization. But, there is no purity percentage, HPLC profile, NMR data, LC-MS, FTIR, melting point, yield percentage, etc..

 Without proper phytochemical characterization, it is difficult to confirm that the observed effects are truly due to stigmasterol.

 Recommendation: The author may include purity analysis, chromatograms, spectral confirmation, extraction yield, and supplementary characterization data from the literature review source.

 3. Dose Justification is Missing. The rationale for selecting 0.25, 0.5, and 0.75 mg/kg BW is not explained.

 Recommendation: authors should explain whether doses were based on previous toxicity studies, pilot experiments, literature references or estimated pharmacological relevance.

 4. Statistical Reporting is Incomplete

 The manuscript reports ANOVA significance but lacks F values, degrees of freedom, exact p-values, effect sizes, confidence intervals.

 For example:

 “p = 0.043”

 is insufficient for high-quality reporting.

 Recommendation: the author need to provide complete statistical reporting following ARRIVE/statistical guidelines.

 5. Network Pharmacology Section is Superficial

 The in silico analysis is descriptive and lacks validation, because there is no molecular docking, no pathway visualization, no experimental validation, and no comparison with reproductive transcriptomic datasets.

 Also, the manuscript inconsistently switches between rat in vivo model, and human protein networks in STRING (“Homo sapiens”).

 Recommendation: the authors should justify the use of human databases, add molecular docking. include KEGG enrichment analysis, and better integrate in vivo findings with network analysis.

 6. Sperm Morphology Methodology Needs Clarification

 The methodology lacks essential details how many sperm cells were counted per animal, whether WHO criteria were adapted, observer blinding procedure, intra/interobserver reliability.

 Recommendation, please provide standardized sperm evaluation criteria.

 7. Overstatement of Contraceptive Potential

 The conclusion:

 “supporting the potential development of stigmasterol as a natural male contraceptive agent”

 is too much because fertility trials were not conducted, reversibility was not assessed, mating success was not tested, and toxicity evaluation was limited.

 Recommendation: please tone down translational claims.

Is the work clearly and accurately presented and does it cite the current literature?

Partly

If applicable, is the statistical analysis and its interpretation appropriate?

Partly

Are all the source data underlying the results available to ensure full reproducibility?

Yes

Is the study design appropriate and is the work technically sound?

Partly

Are the conclusions drawn adequately supported by the results?

Partly

Are sufficient details of methods and analysis provided to allow replication by others?

Partly

Reviewer Expertise:

Pathology Anatomy, Histology, Reproductive Biology, Pharmacology, Toxicology

I confirm that I have read this submission and believe that I have an appropriate level of expertise to confirm that it is of an acceptable scientific standard, however I have significant reservations, as outlined above.

# Response

**Published:** 2026-05-28

**Authors:** Poncojari Wahyono

**Affiliations:** Biology Education, Universitas Muhammadiyah Malang, Malang, East Java, Indonesia

Dear Reviewer,

 We would like to sincerely thank you for the careful evaluation, constructive comments, and valuable recommendations on our manuscript entitled “Stigmasterol from Pluchea indica Leaves Reduces Spermatogenesis and Alters Sperm Morphology in Male Rat: In Vivo and In Silico Study.” We have revised the manuscript thoroughly according to the reviewers’ suggestions.

 All changes in the revised manuscript have been marked in bold, while deleted or replaced text has been indicated using deletion marks where appropriate. The revisions mainly include clarification of the extraction and isolation procedure, addition of solvent and yield information, explanation of sperm collection and sperm morphology assessment procedures, improvement of statistical reporting, more cautious interpretation of hormonal mechanisms, clarification of the in silico analysis, limitation of contraceptive claims, and addition of a study limitations section.

 Below, we provide a point-by-point response to each reviewer’s comments.

 Response to Reviewer 1

 Comment 1. Lack of hormonal measurements

**Reviewer comment:**

*The authors repeatedly conclude that stigmasterol disrupts hormonal regulation and reduces testosterone, LH, and FSH. However, no hormonal assays were conducted. This is a major weakness because the mechanistic conclusions are speculative. The authors should either measure serum testosterone, LH, and FSH levels, or substantially tone down mechanistic claims. Statements such as “Lower testosterone impairs spermatogenesis…” should be framed as hypotheses rather than established findings in this study.*

**Response:**

 We thank the reviewer for this important and scientifically valid comment. We agree that our previous wording overstated the endocrine mechanism because serum testosterone, luteinizing hormone, and follicle-stimulating hormone were not directly measured in the present study.

 Accordingly, we have revised the Abstract, Introduction, Discussion, and Conclusion to substantially tone down all mechanistic claims related to reproductive hormones. We no longer state that stigmasterol directly reduced testosterone, luteinizing hormone, or follicle-stimulating hormone in this study. Instead, we now present hormonal involvement as a possible mechanism or hypothesis based on previous literature and supported indirectly by the predicted involvement of reproductive-related targets in the in silico analysis.

 For example, the revised Discussion now states: “Previous studies have shown that testosterone, luteinizing hormone, and follicle-stimulating hormone are important regulators of spermatogenesis and Sertoli–Leydig cell function. Therefore, a reduction in testosterone or disruption of gonadotropin signaling, if present, could plausibly impair spermatogonial maturation, Sertoli cell support, and spermiogenesis. However, because these reproductive hormones were not directly measured in the present study, this mechanism remains hypothetical.”

 We also added the lack of hormonal assays as a major limitation and recommended that future studies directly measure serum testosterone, luteinizing hormone, follicle-stimulating hormone, inhibin B, and intratesticular testosterone.

**Changes made in the manuscript:**

- Abstract: mechanistic interpretation toned down.
- Introduction: hormonal disruption presented as a possible mechanism.
- Discussion: endocrine mechanism reframed as hypothetical.
- Conclusion: conclusion revised to avoid unsupported hormonal claims.
- Limitations: absence of hormonal assays explicitly acknowledged.

Comment 2. Weak characterization of stigmasterol isolation

**Reviewer comment:**

*The extraction and isolation procedure is inadequately described. The manuscript only mentions chromatography, TLC confirmation, and recrystallization. But there is no purity percentage, HPLC profile, NMR data, LC-MS, FTIR, melting point, yield percentage, etc. Without proper phytochemical characterization, it is difficult to confirm that the observed effects are truly due to stigmasterol.*

**Response:**

 We thank the reviewer for this valuable recommendation. We agree that the previous description of the extraction and isolation process was too brief. We have therefore expanded the subsection “Plant material, extraction, and isolation of stigmasterol” to provide a more detailed and reproducible description.

 The revised manuscript now states that the dried Pluchea indica leaf simplicia was extracted by maceration using methanol as the extraction solvent. We added details on the approximate amount of dried simplicia, solvent ratio, maceration duration, re-maceration process, concentration under reduced pressure, and estimated crude extract yield. We also clarified the fractionation and purification procedure using an n-hexane–ethyl acetate solvent system, silica gel 60 column chromatography, gradient elution, thin-layer chromatography on silica gel 60 F254 plates, visualization under ultraviolet light, detection using Liebermann–Burchard reagent, and recrystallization using methanol.

 We also added the estimated final stigmasterol isolate yield. At the same time, to avoid overclaiming, we explicitly stated that no additional high-performance liquid chromatography, liquid chromatography–mass spectrometry, nuclear magnetic resonance spectroscopy, Fourier-transform infrared spectroscopy, or melting point analysis was performed in the present study. Therefore, the phytochemical characterization is now presented as preliminary and based on chromatographic isolation, thin-layer chromatography confirmation, steroidal reagent detection, and recrystallization.

 This limitation is also acknowledged in the Discussion.

**Changes made in the manuscript:**

- Methods: detailed extraction, fractionation, isolation, TLC, and recrystallization procedure added.
- Methods: extraction yield and isolate yield added.
- Methods: absence of HPLC, LC-MS, NMR, FTIR, and melting point analysis clarified.
- Discussion/Limitations: limited phytochemical characterization acknowledged.

Comment 3. Dose justification is missing

**Reviewer comment:**

*The rationale for selecting 0.25, 0.5, and 0.75 mg/kg BW is not explained. Authors should explain whether doses were based on previous toxicity studies, pilot experiments, literature references, or estimated pharmacological relevance.*

**Response:**

 We thank the reviewer for this helpful comment. We have added a new explanation of dose justification in the Methods section.

 The revised manuscript explains that the doses of 0.25, 0.5, and 0.75 mg/kg body weight were selected by referring to previous preclinical studies on stigmasterol isolated from Pluchea indica leaves in male rats. A prior in vivo antifertility study used the same dose range for 45 days and reported significant changes in sperm concentration and motility. Additional safety-oriented studies using 0.125, 0.25, and 0.5 mg/kg body weight reported no major adverse changes in selected liver enzyme and hematological parameters.

 Based on these references, the present study used 0.25 mg/kg body weight as the low dose, 0.5 mg/kg body weight as the intermediate dose, and 0.75 mg/kg body weight as the higher dose. However, these doses should not be interpreted as established contraceptive doses because fertility trials, mating success assessment, reversibility testing, and comprehensive toxicity evaluation were not conducted in the present study.

**Changes made in the manuscript:**

- Methods: new dose justification text added.
- Discussion/Conclusion: contraceptive-dose interpretation avoided.

Comment 4. Statistical reporting is incomplete

**Reviewer comment:**

*The manuscript reports ANOVA significance but lacks F values, degrees of freedom, exact p-values, effect sizes, and confidence intervals. For example, “p = 0.043” is insufficient for high-quality reporting. The author needs to provide complete statistical reporting following ARRIVE/statistical guidelines.*

**Response:**

 We thank the reviewer for this important methodological recommendation. We have revised the Data analysis and Results sections to improve statistical reporting.

 The revised Data analysis section now states that data were processed using IBM SPSS Statistics software version 26.0. The animal was used as the experimental unit, and each group consisted of six rats. Data were examined for normality using the Shapiro–Wilk test and for homogeneity of variance using Levene’s test. When assumptions were met, one-way analysis of variance was performed, followed by Least Significant Difference post hoc testing when significant.

 We also revised the Results section by adding F values, degrees of freedom, exact p-values, eta squared values, and 95% confidence intervals. For overall spermatogenic cell counts, the revised result is reported as: “Statistical analysis using one-way ANOVA revealed a significant difference in overall spermatogenic cell counts among groups. Based on the reported mean ± standard deviation values and six animals per group, the estimated ANOVA result was F(3,20) = 3.25, exact p = 0.0435, η² = 0.328, indicating a moderate-to-large treatment effect.”

 For sperm morphology, the revised result is reported as: “For normal spermatozoa, the estimated ANOVA result calculated from the reported mean ± standard deviation values was F(3,20) = 55.29, p < 0.001, η² = 0.892. For abnormal spermatozoa, the estimated ANOVA result was F(3,20) = 64.00, p < 0.001, η² = 0.906.”

 We also corrected p-value reporting by replacing p = 0.00 with p < 0.001, because p-values should not be reported as zero.

**Changes made in the manuscript:**

- Methods: “Data analysis” expanded.
- Results: complete ANOVA statistics added.
- Results: effect sizes and confidence intervals added.
- Results: p = 0.00 corrected to p < 0.001.

Comment 5. Network pharmacology section is superficial

**Reviewer comment:**

*The in silico analysis is descriptive and lacks validation because there is no molecular docking, no pathway visualization, no experimental validation, and no comparison with reproductive transcriptomic datasets. Also, the manuscript inconsistently switches between rat in vivo model and human protein networks in STRING (“Homo sapiens”). The authors should justify the use of human databases, add molecular docking, include KEGG enrichment analysis, and better integrate in vivo findings with network analysis.*

**Response:**

 We thank the reviewer for this detailed and constructive comment. We agree that the previous network pharmacology section required clearer scope, stronger justification, and more cautious interpretation.

 We revised the Network pharmacology analysis subsection to clarify that human protein annotations were used because databases such as STRING and DAVID provide more complete and better-curated interaction and enrichment annotations for Homo sapiens than for rat-specific reproductive networks. However, we now explicitly state that the in silico findings require cross-species interpretation and should be considered predictive and hypothesis-generating rather than direct evidence of molecular changes in rat testicular tissue.

 We also clarified that molecular docking was not performed in the present study. Therefore, all wording implying ligand–protein binding validation has been removed or toned down. We added a statement that network pharmacology was used as a target-prediction and pathway-enrichment approach, not as experimental molecular validation.

 Where applicable, we added pathway enrichment interpretation and better integrated the in vivo findings with predicted targets related to steroid signaling, cell proliferation, inflammatory response, and male gonad development. However, we also acknowledged that molecular docking, quantitative polymerase chain reaction, Western blotting, immunohistochemistry, transcriptomic comparison, or other experimental validations are needed in future studies.

**Changes made in the manuscript:**

- Methods: “Network pharmacology analysis” revised.
- Results: in silico findings interpreted more cautiously.
- Discussion: cross-species limitation clarified.
- Discussion/Limitations: absence of molecular docking and experimental validation acknowledged.

Comment 6. Sperm morphology methodology needs clarification

**Reviewer comment:**

*The methodology lacks essential details on how many sperm cells were counted per animal, whether WHO criteria were adapted, observer blinding procedure, and intra/interobserver reliability. Please provide standardized sperm evaluation criteria.*

**Response:**

 We thank the reviewer for this helpful recommendation. We have revised the sperm morphology methodology to provide standardized and more reproducible information.

 The revised manuscript now states that 200 spermatozoa were evaluated for each animal from randomly selected microscopic fields at 400× magnification. We clarified that spermatozoa were classified as normal when the head, midpiece/neck, and tail were intact and structurally continuous. Abnormal spermatozoa were identified based on head defects, detached heads, headless tails, bent or wavy necks, coiled tails, broken tails, angled tails, and other incomplete or distorted structures.

 We also added that these criteria were adapted from standard sperm morphology assessment principles for laboratory animals and World Health Organization-based morphology concepts, with adjustment for rat sperm characteristics.

 To reduce observational bias, we revised the text to state that microscopic slides were coded before evaluation, and the observer assessed sperm morphology without knowing the treatment group. We also clarified that the same observer evaluated all slides using identical criteria to maintain intra-observer consistency. Because the slides were not independently evaluated by a second observer, formal inter-observer reliability statistics were not calculated. This limitation has been acknowledged in the Discussion.

**Changes made in the manuscript:**

- Methods: number of spermatozoa evaluated per animal added.
- Methods: standardized normal and abnormal morphology criteria added.
- Methods: observer blinding/coded slides clarified.
- Discussion/Limitations: absence of formal inter-observer reliability acknowledged.

Comment 7. Overstatement of contraceptive potential

**Reviewer comment:**

*The conclusion “supporting the potential development of stigmasterol as a natural male contraceptive agent” is too much because fertility trials were not conducted, reversibility was not assessed, mating success was not tested, and toxicity evaluation was limited. Please tone down translational claims.*

**Response:**

 We thank the reviewer for this important comment. We agree that the previous conclusion overstated the translational implications of the study. We have therefore revised the Abstract, Discussion, and Conclusion to avoid claiming that stigmasterol is ready to be developed as a natural male contraceptive agent.

 The revised Discussion now states: “Taken together, the findings suggest that stigmasterol exposure was associated with reduced spermatogenic cell counts and increased sperm abnormalities in male rats. The in silico analysis further generated plausible molecular hypotheses involving steroid signaling, cell proliferation, and inflammatory pathways. However, the present study does not provide sufficient evidence to support direct contraceptive development claims. Further studies involving fertility trials, mating success assessment, reversibility testing, reproductive hormone assays, systemic toxicity evaluation, and stronger phytochemical characterization are required before stigmasterol can be considered a validated male contraceptive candidate.”

 The revised Conclusion also states that the findings should be interpreted as preliminary preclinical evidence rather than definitive proof of male contraceptive efficacy.

**Changes made in the manuscript:**

- Abstract: contraceptive claim toned down.
- Discussion: translational claim revised.
- Conclusion: direct contraceptive development claim removed.
- Limitations: fertility trials, reversibility, mating success, and toxicity limitations added.

Closing Statement

 We sincerely appreciate the reviewers’ constructive comments, which have helped us improve the clarity, methodological transparency, statistical reporting, and scientific interpretation of the manuscript. We believe that the revised version is now more cautious, better aligned with the measured parameters, and more transparent regarding the limitations of the study.

 Thank you for your valuable time and consideration.

 Sincerely,

 The Authors
